# Functional Regression Models for Epistasis Analysis of Multiple Quantitative Traits

**DOI:** 10.1371/journal.pgen.1005965

**Published:** 2016-04-22

**Authors:** Futao Zhang, Dan Xie, Meimei Liang, Momiao Xiong

**Affiliations:** 1 Department of Computer Science, College of Internet of Things, Hohai University, Changzhou, China; 2 College of Information Engineering, Hubei University of Chinese Medicine, Hubei, China; 3 Institute of Bioinformatics, Zhejiang University, Hangzhou, Zhejiang, China; 4 Human Genetics Center, Division of Biostatistics, The University of Texas School of Public Health, Houston, Texas, United States of America; Case Western Reserve University School of Medicine, UNITED STATES

## Abstract

To date, most genetic analyses of phenotypes have focused on analyzing single traits or analyzing each phenotype independently. However, joint epistasis analysis of multiple complementary traits will increase statistical power and improve our understanding of the complicated genetic structure of the complex diseases. Despite their importance in uncovering the genetic structure of complex traits, the statistical methods for identifying epistasis in multiple phenotypes remains fundamentally unexplored. To fill this gap, we formulate a test for interaction between two genes in multiple quantitative trait analysis as a multiple functional regression (MFRG) in which the genotype functions (genetic variant profiles) are defined as a function of the genomic position of the genetic variants. We use large-scale simulations to calculate Type I error rates for testing interaction between two genes with multiple phenotypes and to compare the power with multivariate pairwise interaction analysis and single trait interaction analysis by a single variate functional regression model. To further evaluate performance, the MFRG for epistasis analysis is applied to five phenotypes of exome sequence data from the NHLBI’s Exome Sequencing Project (ESP) to detect pleiotropic epistasis. A total of 267 pairs of genes that formed a genetic interaction network showed significant evidence of epistasis influencing five traits. The results demonstrate that the joint interaction analysis of multiple phenotypes has a much higher power to detect interaction than the interaction analysis of a single trait and may open a new direction to fully uncovering the genetic structure of multiple phenotypes.

## Author Summary

The widely used statistical methods test interaction for single phenotype. However, we often observe pleotropic genetic interaction effects. The simultaneous gene-gene (GxG) interaction analysis of multiple complementary traits will increase statistical power to detect GxG interactions. Although GxG interactions play an important role in uncovering the genetic structure of complex traits, the statistical methods for detecting GxG interactions in multiple phenotypes remains less developed owing to its potential complexity. Therefore, we extend functional regression model from single variate to multivariate for simultaneous GxG interaction analysis of multiple correlated phenotypes. Large-scale simulations are conducted to evaluate Type I error rates for testing interaction between two genes with multiple phenotypes and to compare power with traditional multivariate pair-wise interaction analysis and single trait interaction analysis by a single variate functional regression model. To further evaluate performance, the MFRG for interaction analysis is applied to five phenotypes of exome sequence data from the NHLBI’s Exome Sequencing Project (ESP) to detect pleiotropic GxG interactions. 267 pairs of genes that formed a genetic interaction network showed significant evidence of interactions influencing five traits.

## Introduction

In the past several years, we have witnessed remarkable progresses in the development of methodologies for identification of epistasis that detect deviation from summation of genetic additive effects for a quantitative trait [1]. The classical approach to epistasis analysis is a single variant test. The epistasis is typically evaluated by testing interaction between a pair of variants one at a time. The classical methods for epistasis tests are originally designed to detect epistasis for common variants and are difficult applied to rare variants due to multiple testing problems and the low power to detect interaction. To overcome the critical barrier in interaction analysis for rare variants, instead of testing each pair of variants individually, group interaction tests that evaluate cumulative interaction effects of multiple genetic variants in a region or gene have recently been developed. Regression-based methods [2–8], haplotype-based methods [9–15], and machine learning-based methods [16–20] are proposed for epistasis analysis.

The classical statistical methods for interaction analysis have mainly tested association with single traits, one time analyzing one trait [21]. However, multiple phenotypes are highly correlated. More than 4.6% of the SNPs and 16.9% of the genes in previous genome-wide association studies (GWAS) are reported to be significantly associated with more than one trait [22]. These results demonstrate that genetic pleiotropic effects likely play a crucial role in the molecular basis of correlated phenotypes [23–26]. Joint epistasis analysis of multiple complementary traits will increase statistical power to unravel the interaction structure of multiple phenotypes [27, 28]. Despite their importance in understanding genetic mechanism underlying the complex diseases, the statistical methods for identifying epistasis in multiple phenotypes have been less developed [1]. The interaction analyses for multiple phenotypes have been limited to common variants in carefully controlled experimental crosses [29, 30]. Simultaneously analyzing interactions for multiple phenotypes in humans poses enormous challenges for methodologies and computations.

Purpose of this paper is to develop a general analytic framework and novel statistical methods for simultaneous epistasis analysis of multiple correlated phenotypes. To unify the approach to epistasis analysis for both common and rare variants, we take a genome region (or gene) as a basic unit of interaction analysis and use all the information that can be accessed to collectively test interaction between all possible pairs of SNPs within two genome regions (or genes). Functional data analysis is used to reduce the dimension of next-generation sequencing data. Specifically, genetic variant profiles that will recognize information contained in the physical location of the SNP are used as a major data form. The densely typed genetic variants in a genomic region for each individual are so close that these genetic variant profiles can be treated as observed data taken from curves [8, 31]. Since standard multivariate statistical analyses often fail with functional data [32] we formulate a test for interaction between two genomic regions in multiple quantitative trait analysis as a multiple functional regression (MFRG) model [33] with scalar response. In the MFRG model the genotype functions (genetic variant profiles) are defined as a function of the genomic position of the genetic variants rather than a set of discrete genotype values and the quantitative trait is predicted by genotype functions with their interaction terms. By functional principal component analysis, the genotype functions are expanded as a few functional principal components (FPC) and the MFRG model is transformed to the classical multivariate regression model (MRG) in which FPC scores are taken as variates. Statistics are developed in this publication which can be applied to pairwise interaction tests and gene-based interaction tests for multiple phenotypes. By investigating SNP-SNP interactions or gene-gene interactions that are shared across multiple traits, pleiotropic epistasis can be studied.

To evaluate performance for multiple traits epistasis analysis, large scale simulations are used to calculate the Type I error rates of the MFRG for testing interaction between two genomic regions with multiple phenotypes and to compare power with multivariate pair-wise interaction analysis and single trait interaction analysis by functional regression (FRG) model. To further evaluate performance, the MFRG for epistasis analysis is applied to five traits: high density lipoprotein (HDL), low density lipoprotein (LDL), total cholesterol, systolic blood pressure (SBP), and diastolic blood pressure (DBP), from exome sequence data from the NHLBI’s Exome Sequencing Project (ESP) to detect pleiotropic epistasis.

## Methods

Assume that *n* individuals are sampled. Let *y*_*ik*_, *k* = 1,2,…,*K*, be the *k*-th trait values of the *i*-th individual. Consider two genomic regions [*a*_1_, *b*_1_] and [*a*_2_, *b*_2_]. Let *x*_*i*_(*t*) and *x*_*i*_(*s*) be genotypic functions of the *i*-th individual defined in the regions [*a*_1_, *b*_1_] and [*a*_2_, *b*_2_], respectively. Let *y*_*i*_ = [*y*_*i*1_,…,*y*_*iK*_]^*T*^ be the vector of the trait values measured on the *i*-th individual. Let *t* and *s* be a genomic position in the first and second genomic regions, respectively. Define a genotype profile *x*_*i*_(*t*) of the i-th individual as

$$X_{i}(t)=\{\begin{array}{c} 0,\;\text{mm}\\ 1,\;\text{Mm}\\ 2,\;\text{MM} \end{array},$$

where M and m are two alleles of the marker at the genomic position *t*. Recall that a regression model for interaction analysis with the *k*-th trait is defined as

$$y_{ik}=\mu_{k}+\sum_{d=1}^{D}\nu_{id}\tau_{kd}+\sum_{j=1}^{J_{1}}x_{ij}\alpha_{kj}+\sum_{l=1}^{J_{2}}z_{il}\beta_{kl}+\sum_{j=1}^{J_{1}}\sum_{l=1}^{J_{2}}x_{ij}z_{il}\gamma_{kjl}+\varepsilon_{ik},$$ (1)

where μ_*k*_ is an overall mean of the *k*-th trait, *ζ*_*kd*_ is the coefficient associated with the covariate ***ν***_*d*_, α_*kj*_ is the main genetic additive effect of the *j*-th SNP in the first genomic region for the *k*-th trait, β_*kl*_ is the main genetic additive effect of the *l*-th SNP in the second genomic region for the *k*-th trait, γ_*kjl*_ is an additive × additive interaction effect between the *j*-th SNP in the first genomic region and the *l*-th SNP in the second genomic region for the *k*-th trait; *x*_*ij*_ and *z*_*il*_ are indicator variableS for the genotypes at the *j*-th SNP and the *l*-th SNP, respectively; ε_*ik*_, *k* = 1,..,*K* are independent and identically distributed normal variables with mean of zero and covariance matrix Σ.

Similar to the multiple regression models for interaction analysis with multiple quantitative traits, the functional regression model for a quantitative trait can be defined as

$$y_{ik}=\alpha_{0k}+\sum_{d=1}^{D}\nu_{id}\tau_{kd}+\int_{T}\alpha_{k}(t)x_{i}(t)dt+\int_{S}\beta_{k}(s)x_{i}(s)ds+\int_{T}\int_{S}\gamma_{k}(t,s)x_{i}(t)x_{i}(s)dtds+\varepsilon_{i}{}_{k},$$ (2)

where α_0*k*_ is an overall mean, *ζ*_*kd*_ is defined as before, *α*_*k*_(*t*) and *β*_*k*_(*s*) are genetic additive effects of two putative QTLs located at the genomic positions *t* and *s*, respectively; γ_*k*_(*t*,*s*) is the interaction effect between two putative QTLs located at the genomic positions *t* and *s* for the *k*-th trait, *k* = 1,…,*K*, *x*_*i*_(*t*) and *x*_*i*_(*s*) are genotype profiles, and ε_*ik*_ are independent and identically distributed normal variables with mean of zero and covariance matrix Σ. Consider covariates in the model (2) allows incorporating PCA scores for population stratification, sex, age, BMI and other biomarkers into the model.

### Estimation of Interaction Effects

We assume that both phenotypes and genotype profiles are centered. The genotype profiles *x*_*i*_(*t*) and *x*_*i*_(*s*) are expanded in terms of the orthonormal basis function as:

$$x_{i}(t)=\sum_{j=1}^{\infty }\xi_{ij}\phi_{j}(t)\;\text{and}$$

$$x_{i}(s)=\sum_{l=1}^{\infty }\eta_{il}\psi_{l}(s),$$ (3)

where ϕ_*j*_(*t*) and ψ_*l*_(*s*) are sequences of the orthonormal basis functions. The more number of variants in the genes the more accurate the eigenfunction expansion. If the number of variants is less than 3 the eigenfunction expansion of the genotypic profiles is impossible. MFRG can only be used for gene with more than 3 variants.

In practice, numerical methods for the integral will be used to calculate the expansion coefficients. Substituting Eq (3) into Eq (2), we obtain (Appendix)

$$y_{ik}=\alpha_{0k}+\sum_{d=1}^{D}\nu_{id}\tau_{kd}+\sum_{j=1}^{\infty }\xi_{ij}\alpha_{kj}+\sum_{l=1}^{\infty }\eta_{il}\beta_{kl}+\sum_{j=1}^{\infty }\sum_{l=1}^{\infty }\xi_{ij}\eta_{il}\gamma_{kjl}+\varepsilon_{ik},i=1,\ldots ,n,k=1,\ldots ,K,$$ (4)

The parameters *α*_*kj*_, *β*_*kl*_ and *γ*_*kjl*_ are referred to as genetic additive and additive × additive effect scores for the *k*-th trait. These scores can also be viewed as the expansion coefficients of the genetic effect functions with respect to orthonormal basis functions.

Then, Eq (4) can be approximated by (Appendix)

$$\begin{array}{l} Y=e\alpha_{0}+\nu \tau +\xi \alpha +\eta \beta +\Gamma \gamma +\varepsilon \\ \;=WB+\varepsilon , \end{array}$$ (5)

where $W=\left[\begin{array}{ccccc} e & \nu & \xi & \eta & \Gamma \end{array}\right]$ and $B=\left[\begin{array}{c} \alpha_{0}\\ \tau \\ \alpha \\ \beta \\ \gamma \end{array}\right]$.

Therefore, we transform the original functional regression interaction model into the classical multivariate regression interaction model by eigenfunction expansions. All methods for multivariate regression interaction analysis can directly be used for solving problem (5).

The standard least square estimators of B and the variance covariance matrix Σ are, respectively, given by

$$\hat{B}={\left(W^{T}W\right)}^{-1}W^{T}Y,$$ (6)

$$\hat{\Sigma }=\frac{1}{n}{\left(Y-WB\right)}^{T}(Y-WB).$$ (7)

Denote the last *JL* row of the matrix (*W*^*T*^*W*)^−1^*W*^*T*^ by *A*. Then, the estimator of the parameter γ is given by

$$\hat{\gamma }=AY.$$ (8)

The vector of the matrix γ can be written as

$$vec(\hat{\gamma })=(I⊗A)vec(Y).$$ (9)

By the assumption of the variance matrix of *Y*, we obtain the variance matrix of *vec*(*Y*):

var(vec(Y))=Σ⊗I. (10)

Thus, it follows from Eqs (9) and (10) that

$$\begin{array}{l} \Lambda \;=\text{var}(vec(\hat{\gamma }))=(I⊗A)(\Sigma ⊗I)(I⊗A^{T})\\ \;=\Sigma ⊗(AA^{T}). \end{array}$$ (11)

### Test Statistics

An essential problem in genetic interaction studies of the quantitative traits is to test the interaction between two genomic regions (or genes). Formally, we investigate the problem of testing the following hypothesis:

$$\gamma_{k}(t,s)=0,\forall t\in [a_{1},b_{1}],s\in [a_{2},b_{2}],k=1,\ldots ,K,$$

which is equivalent to testing the hypothesis:

$$H_{0}\;:\;\gamma =0.$$

Define the test statistic for testing the interaction between two genomic regions [*a*_1_, *b*_1_] and [*a*_2_, *b*_2_] with *K* quantitative traits as

$$T_{I}={\left(vec(\hat{\gamma })\right)}^{T}\;\Lambda^{-1}vec(\hat{\gamma }).$$ (12)

Then, under the null hypothesis *H*_0_: γ = 0, *T*_*I*_ is asymptotically distributed as a central $\chi_{\left(KJL\right)}^{2}$ distribution if *JL* components are taken in the expansion Eq (3).

Group tests often make implicit homogeneity assumptions where all putatively functional variants within the same genomic region are assumed to have the same direction of effects. However, in practice, the variants with opposite directions of effects will be simultaneously presented in the same genomic region. MFRG can efficiently use information of both risk and protective variants and allow for sign and size heterogeneity of genetic variants. In general, the trait increasing and decreasing variants will be present in different locations in the genomic region. Information of trait increasing and decreasing variants usually will be reflected in different eigenfunctions and hence will be included in different functional principal component scores. The MFRG test statistic is essentially to summarize the square of the functional principal component scores. Therefore, the opposite effects of trait increasing and decreasing variants on the phenotype will not compromise each other in the MFRG test statistics. The MFRG statistics automatically take the opposite effects of the trait increasing and decreasing variants on the phenotype into account and do not require additional computations. MFRG will take the sign and size heterogeneity of the variants into account and be less sensitive to the presence of variants with opposite directions of effect.

We can also develop likelihood ratio-based statistics for testing interaction.

Setting $W=\left[\begin{array}{cc} W_{1} & W_{2} \end{array}\right]$, we can write the model as

$$E[Y]=W_{1}\left[\begin{array}{c} \alpha \\ \beta \end{array}\right]+W_{2}\gamma .$$

Under *H*_0_: γ = 0, we have the model:

$$Y=W_{1}\left[\begin{array}{c} \alpha \\ \beta \end{array}\right]+\varepsilon .$$

The estimators will be

$$\left[\begin{array}{c} \hat{\alpha }\\ \hat{\beta } \end{array}\right]={\left(W_{1}^{T}W_{1}\right)}^{-1}W_{1}^{T}Y\;\text{and}\;{\hat{\Sigma }}_{1}=\frac{1}{n}{\left(Y-W_{1}\left[\begin{array}{c} \hat{\alpha }\\ \hat{\beta } \end{array}\right]\right)}^{T}(Y-W_{1}\left[\begin{array}{c} \hat{\alpha }\\ \hat{\beta } \end{array}\right]).$$

The likelihood for the full model and reduced model are, respectively, given by

$$L(\hat{\alpha },\hat{\beta },\hat{\gamma },\hat{\Sigma })=\frac{e^{-nK/2}}{{\left(2\pi \right)}^{nK/2}|\hat{\Sigma }{|}^{n/2}}\;\text{and}$$

$$L(\hat{\alpha },\hat{\beta },{\hat{\Sigma }}_{1})=\frac{e^{-nK/2}}{{\left(2\pi \right)}^{nK/2}|{\hat{\Sigma }}_{1}{|}^{n/2}}.$$

The likelihood-ratio-based statistic for testing interaction between two genomic regions with multivariate traits is defined as

$$T_{I\Lambda }=-n\;\text{log}\left(\frac{\left|\hat{\Sigma }\right|}{\left|{\hat{\Sigma }}_{1}\right|}\right).$$ (13)

Under the null hypothesis *H*_0_: γ = 0, *T*_*I*Λ_ is asymptotically distributed as a central $\chi_{\left(KJL\right)}^{2}$ distribution if *JL* components are taken in the expansion Eq (3).

### Simulation Model for Type 1 Error Rate Calculation

The genetic models for simulations to calculate Type 1 error rates of the tests are briefly given below. We first assume the model with no marginal effects for all traits:

$$Y_{i}=\mu +\varepsilon_{i},\;i=1,\ldots ,n,$$

where *Y*_*i*_ = [*y*_*i*1_,…,*y*_*ik*_], *μ* = [*μ*_1,_…,*μ*_*k*_], and *ε*_*i*_ is distributed as

$$\left[\begin{array}{ccc} \varepsilon_{1} & \ldots & \varepsilon_{k} \end{array}\right]∼N\left(\left[\begin{array}{ccc} 0 & \ldots & 0 \end{array}\right],\left(\begin{array}{ccc} 1 & \cdots & 0.5\\ \vdots & \ddots & \vdots \\ 0.5 & \cdots & 1 \end{array}\right)\right).$$

Then, we considered the model with marginal genetic effect (additive model) at one gene:

$$y_{ik}=\mu_{k}+\sum_{j=1}^{J}x_{ij}\alpha_{kj}+\varepsilon_{ik},$$

where

$$x_{ij}=\left\{ \begin{array}{cc} 2(1-P_{j}) & A_{j}A_{j}\\ 1-2P_{j} & A_{j}a_{j}\\ -2P_{j} & a_{j}a_{j} \end{array}\right.,\;\alpha_{k}=(r_{k}-1)f_{0},$$

where *P*_*j*_ is a frequency of the allele *A*_*j*_, *r*_*k*_ is a risk parameter of the *k*-th trait which was randomly selected from 1.1 to 1.6. The risk parameter affect the genetic effects and is used to control the contribution effort by genotype to the phenotype. The risk parameter influences the relative magnitude of the genetic effects. *f*_0_ is a baseline penetrance and set to 1 and *ε* are defined as before.

Finally, we consider the model with marginal genetic effects (additive model) at both genes:

$$y_{ik}=\mu_{k}+\sum_{j=1}^{J}x_{ij}\alpha_{kj}+\sum_{l=1}^{L}z_{il}\beta_{kl}+\varepsilon_{ik},$$

where

$$x_{ij}=\left\{ \begin{array}{cc} 2(1-P_{j}) & \;A_{j}A_{j}\\ 1-2P_{j} & \;A_{j}a_{j}\\ -2P_{j} & a_{j}a_{j} \end{array}\right.\;,\;{\text{z}}_{il}=\{\begin{array}{cc} 2(1-q_{l}) & B_{l}B_{l}\\ 1-2q_{l} & B_{l}b_{l}\\ -2q_{l} & b_{l}b_{l} \end{array}\;,\;\alpha_{kj}=\alpha_{k}=(r_{pk}-1)f_{0},\beta_{kl}=\beta_{k}=(r_{qk}-1)f_{0},$$

*P*_*j*_ and *q*_*l*_ are frequencies of the alleles *A*_*j*_ and *B*_*l*_, respectively, *r*_*pk*_ and *r*_*qk*_ are risk parameters of the *k*-th trait for the SNPs in the first and second genes, respectively, and randomly selected from 1.1 to 1.6, *f*_0_ is a baseline penetrance and set to 1 and *ε* are defined as before.

## Results

### Null Distribution of Test Statistics

To examine the null distribution of test statistics, we performed a series of simulation studies to compare their empirical levels with the nominal ones. We calculated the Type I error rates for rare alleles, and common alleles. To make simulations more close to real whole exome sequencing data, we generated 50,000 datasets consisting of 1,000,000 chromosomes randomly sampled from the NHLBI’s Exome Sequencing Project (ESP) with 2,016 individuals and 18,587 genes. Each dataset included randomly selected a pair of genes from sequenced 18,587 genes. We randomly selected 20% of SNPs from each gene as causal variants. The number of sampled individuals from populations of 1,000,000 chromosomes ranged from 1,000 to 5,000. For each dataset, we repeated 5,000 simulations. We presented average type I error rates over 50,000 randomly selected pairs of genes from whole exome sequencing ESP dataset.

Table 1 and S1 and S2 Tables summarized the average Type I error rates of the test statistics for testing the interaction between two genes with no marginal effect and consisting of only rare variants with 5 traits, 2 traits and 10 traits, respectively, over 50,000 pairs of genes at the nominal levels α = 0.05, α = 0.01 and α = 0.001. Table 2 and S3 and S4 Tables summarized the average Type I error rates of the test statistics for testing the interaction between two genes with marginal effect at one gene consisting of only rare variants with 5 traits, 2 traits and 10 traits, respectively, over 50,000 pairs of genes at the nominal levels α = 0.05, α = 0.01 and α = 0.001. Table 3 and S5 and S6 Tables summarized the average Type I error rates of the test statistics for testing the interaction between two genes with marginal effect at both genes consisting of only rare variants with 5 traits, 2 traits and 10 traits, respectively, over 50,000 pairs of genes at the nominal levels α = 0.05, α = 0.01 and α = 0.001. For common variants, we summarized the average Type I error rates of the test statistics for testing the interaction between two genes with marginal effect at both genes consisting of only common variants with 5 traits, 2 and 10 traits, respectively, over 10 pairs of genes at the nominal levels α = 0.05, α = 0.01 and α = 0.001, in Table 4 and S7 and S8 Tables, respectively. The statistics for testing interaction between two genomic regions with only common variants have the similar Type 1 error rates in the other two scenarios: with marginal genetic effects at one gene or without marginal genetic effects at two genes. These results clearly showed that the Type I error rates of the MFRG-based test statistics for testing interaction between two genes with multiple traits and common variants with or without marginal effects were not appreciably different from the nominal α levels. For the rare variants when the sample sizes increased to 5,000, the Type 1 error rates were still not appreciably different from the nominal levels.

**Table 1:** Average type 1 error rates of the statistic for testing interaction between two genes with no marginal effect consisting only rare variants with 5 traits over random selected 50,000 pairs of genes from whole exome.

Sample Size	0.05	0.01	0.001
1000	0.0784	0.0188	0.0019
2000	0.0693	0.0097	0.0016
3000	0.0617	0.0135	0.0010
4000	0.0591	0.0126	0.0014
5000	0.0546	0.0095	0.0012

**Table 2:** Average type 1 error rates of the statistic for testing interaction between two genes with marginal effect at one gene consisting only rare variants with 5 traits over randomly selected 50,000 pairs of genes from the whole exome.

Sample Size	0.05	0.01	0.001
1000	0.0785	0.0177	0.0018
2000	0.0672	0.0154	0.0017
3000	0.0604	0.0149	0.0010
4000	0.0555	0.0120	0.0012
5000	0.0510	0.0132	0.0009

**Table 3:** Average type 1 error rates of the statistic for testing interaction between two genes with marginal effects at two genes consisting only rare variants with 5 traits over randomly selected 50,000 pairs of genes from the whole exome.

Sample Size	0.05	0.01	0.001
1000	0.0715	0.0152	0.0018
2000	0.0664	0.0133	0.0013
3000	0.0596	0.0105	0.0014
4000	0.0508	0.0098	0.0010
5000	0.0511	0.0106	0.0012

**Table 4:** Average type 1 error rates of the statistic for testing interaction between two genes with marginal effects at two genes consisting only common variants with 5 traits over randomly selected 50,000 pairs of genes from the whole exome.

Sample Size	0.05	0.01	0.001
1000	0.0529	0.0112	0.0011
2000	0.0513	0.0098	0.0013
3000	0.0499	0.0101	0.0009
4000	0.0471	0.0094	0.0010
5000	0.0469	0.0102	0.0008

### Power Evaluation

To evaluate the performance of the MFRG models for interaction analysis of multiple traits, we used simulated data to estimate their power to detect interaction between two genes for two, four, five, six and ten quantitative traits. A true multiple quantitative genetic model is given as follows. Consider *H* pairs of quantitative trait loci (QTL) from two genes (genomic regions). Let $Q_{h_{1}}$ and $q_{h_{1}}$ be two alleles at the first QTL, and $Q_{h_{2}}$ and $q_{h_{2}}$ be two alleles at the second QTL, for the *H* pair of QTLs. Let *u*_*ijkl*_ be the genotypes of the *u*-th individual with $ij=Q_{h_{1}}Q_{h_{1}},Q_{h_{1}}q_{h_{1}},q_{h_{1}}q_{h_{1}}$ and $kl=Q_{h_{2}}Q_{h_{2}},Q_{h_{2}}q_{h_{2}},q_{h_{2}}q_{h_{2}}$, and $g_{mu_{ijkl}}$ be its genotypic value for the *m*-th trait. The following multiple regression is used as a genetic model for the *m*-th quantitative trait:

$$y_{mu}=\sum_{h=1}^{H}g_{mu_{ijkl}}^{h}+\varepsilon_{mu}\;,\;u=1,2,\ldots ,n,m=1,\ldots ,M,$$

where $g_{mu_{ijkl}}^{h}$ is a genotypic value of the *h*-th pair of QTLs for the *m*-th quantitative trait and *ε*_*mu*_ are distributed as $\left[\begin{array}{ccc} \varepsilon_{1} & \ldots & \varepsilon_{\text{m}} \end{array}\right]∼N\left(\left[\begin{array}{ccc} 0 & \ldots & 0 \end{array}\right],\left(\begin{array}{ccc} 1 & \cdots & 0.5\\ \vdots & \ddots & \vdots \\ 0.5 & \cdots & 1 \end{array}\right)\right)$.

Four models of interactions are considered: (1) Dominant OR Dominant, (2) Dominant AND Dominant, (3) Recessive OR Recessive and (4) Threshold model (S9 Table). We assume that the genotypes at two loci affect a complex trait. Intuitively, Dominant OR Dominant model means that presence of risk allele at least one locus will cause the phenotype variation. Dominant AND Dominant model means that only when risk alleles at both loci are present the phenotype variation can be affected. Recessive OR recessive model indicates that when both risk alleles are at least present at one locus the phenotype variation can be observed. Threshold model implies that when two risk alleles at one locus and at least one risk allele at another locus are present, the phenotype variation will be observed. Recessive AND Recessive model is excluded due to low frequency of that condition with rare variants. The risk parameter *r* varies from 0 to 1.

We generated 2,000,000 chromosomes by resampling from 2,016 individuals of European origin with variants in random two genes selected from the NHLBI’s Exome Sequencing Project (ESP). Two haplotypes were randomly sampled from the population and assigned to an individual. We randomly selected 20% of the variants as causal variants. A total of 2,000 individuals for the four interaction models were sampled from the populations. A total of 1,000 simulations were repeated for the power calculation.

The power of the proposed MFRG model is compared with the single trait functional regression (SFRG) model, the multi-trait pair-wise interaction test and the regression on principal components (PCs). For SNPs genotypes in each genomic region principal component analysis (PCA) were performed. The number of principal components for each individual which can explain 80% of the total genetic variation in the genomic region will be selected as the variables. Specifically, the principal component score of the i-th individual in the first and second genomic regions are denoted by $x_{i1},\ldots ,x_{ik_{1}}$ and $z_{i1},\ldots z_{ik_{2}}$, respectively. The regression model for detection of interaction for the m-th trait is then given by

$$y_{mi}=\mu_{m}+\sum_{j=1}^{k_{1}}x_{ij}\alpha_{mj}+\sum_{l=1}^{k_{2}}z_{il}\beta_{ml}+\sum_{j=1}^{k_{1}}\sum_{l=1}^{k_{2}}x_{ij}z_{il}\gamma_{mjl}+\varepsilon_{mi}.$$

The power of the MFRG is compared with the traditional point-wise interaction test which takes the following model:

$$y_{mi}=\mu_{m}+x_{i1}\alpha_{m1}+x_{i2}\alpha_{m2}+x_{i1}x_{i2}\gamma_{m}+\varepsilon_{mi},i=1,\ldots ,n,m=1,\ldots ,M.$$

For a pair of genes, we assume that the first gene has *k*_1_ SNPs, and the second gene has *k*_2_ SNPs, then, the total number of all possible pairs is *k* = *k*_1_ × *k*_2_. For each pair of SNPs, we calculated a statistic for testing pair-wise interaction *T*_mjpair_. Finally, the maximum of *T*_mjpair_: *T*_max_ = max(*T*_1,1pair_,*T*_1,2pair_,…,*T*_1,kpair_,…,*T*_*M*,1pair_,…,*T*_*M*,kpair_) is computed.

**Fig 1:**
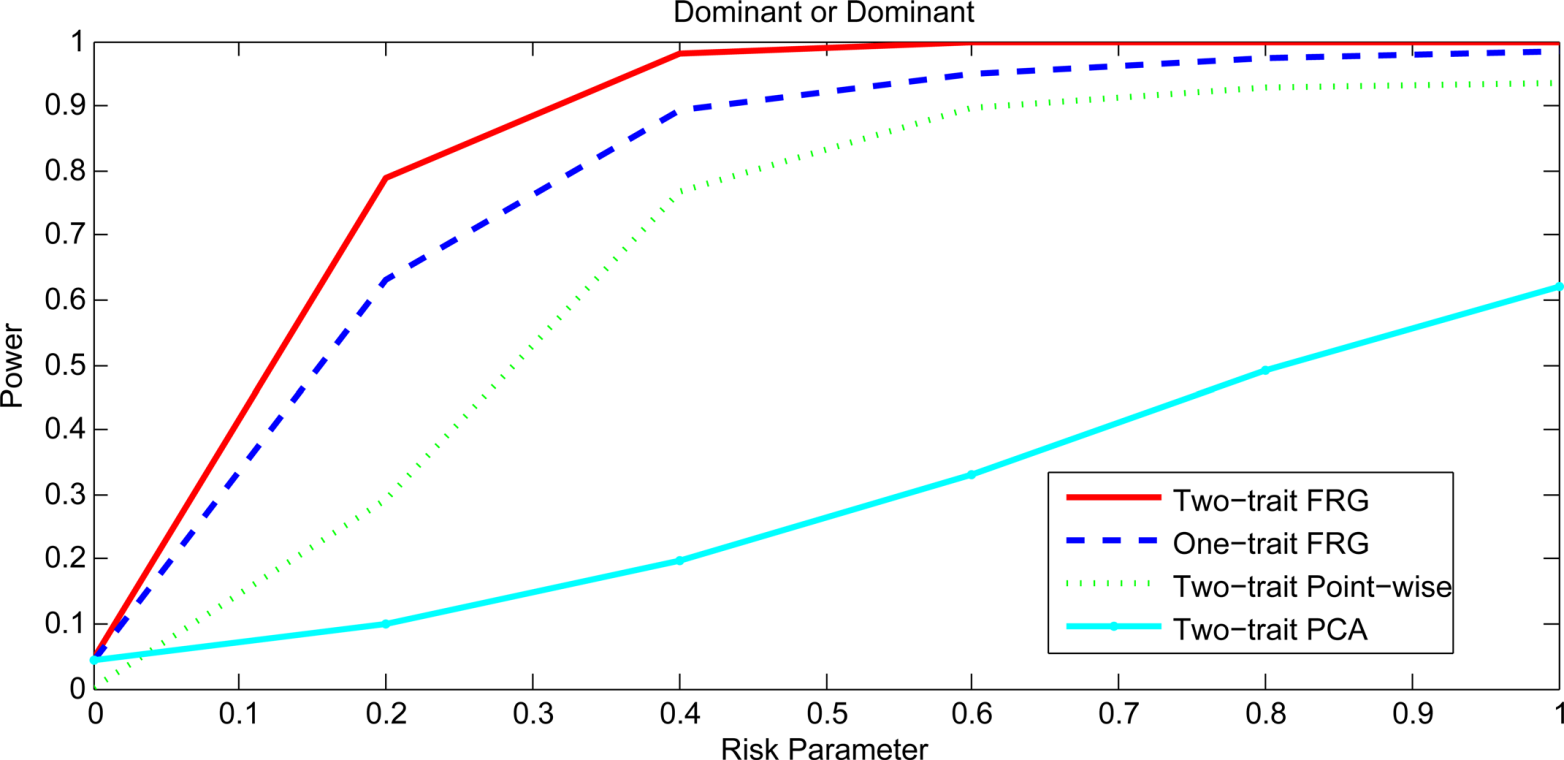
Power curves under Dominant OR Dominant with two genes including rare variants only.

**Fig 2:**
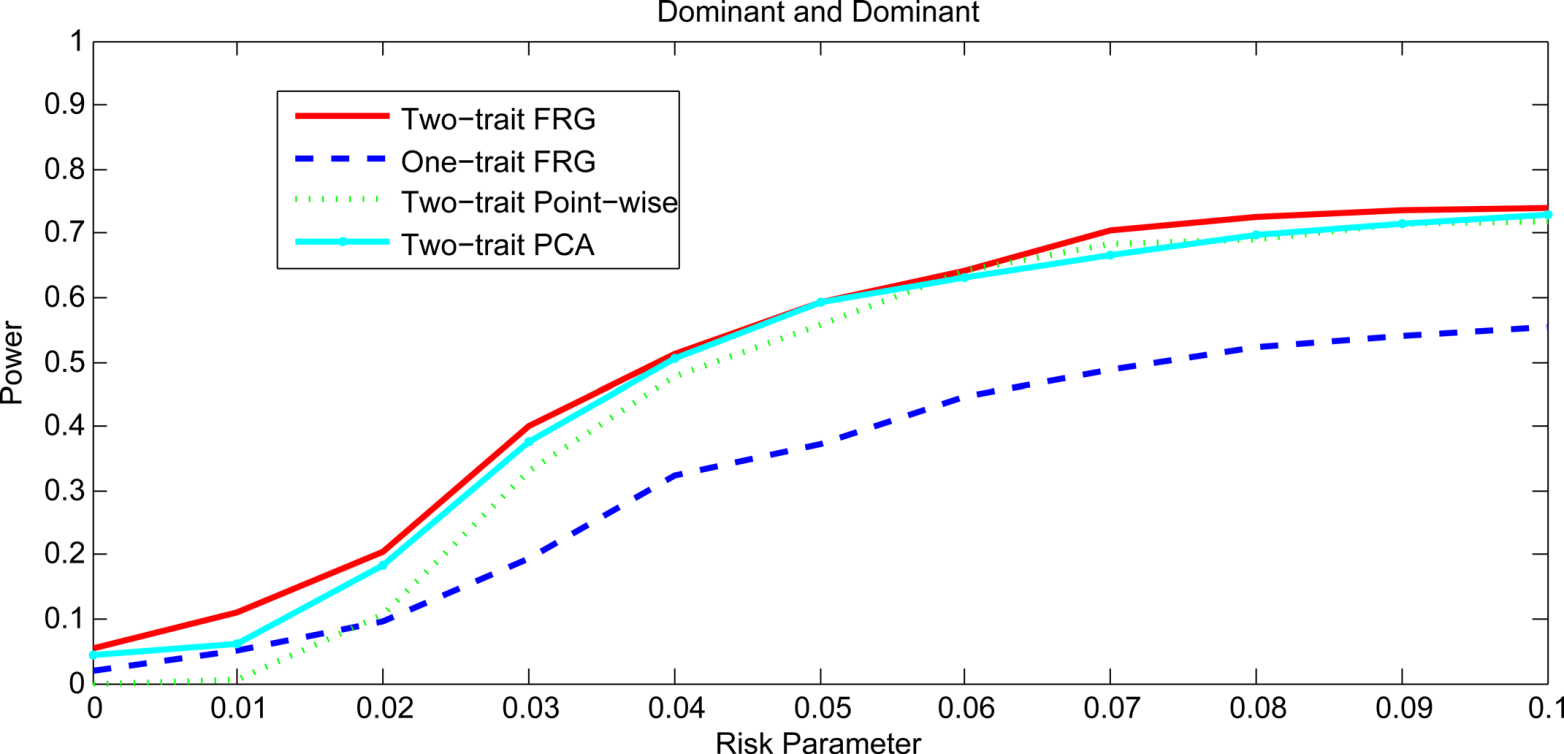
Power curves under Dominant AND Dominant with two genes including rare variants only.

Figs 1 and 2, S1 Fig and S2 Fig plotted the power curves of the two-trait FRG, single trait FRG, two-trait regression on PCs and two-trait pair-wise interaction tests for a quantitative trait under Dominant OR Dominant, Dominant AND Dominant, Threshold, and Recessive OR Recessive models, respectively. Only two genes include rare variants. These power curves are a function of the risk parameter at the significance level α = 0.05. Permutations in the point-wise interaction tests were used to adjust for multiple testing. In all cases, the two-trait FRG had the highest power to detect epistasis. We observed two remarkable features. First, two-trait test had higher power than the one-trait test. Second, the two-trait FRG had the highest power among all two-trait tests.

**Fig 3:**
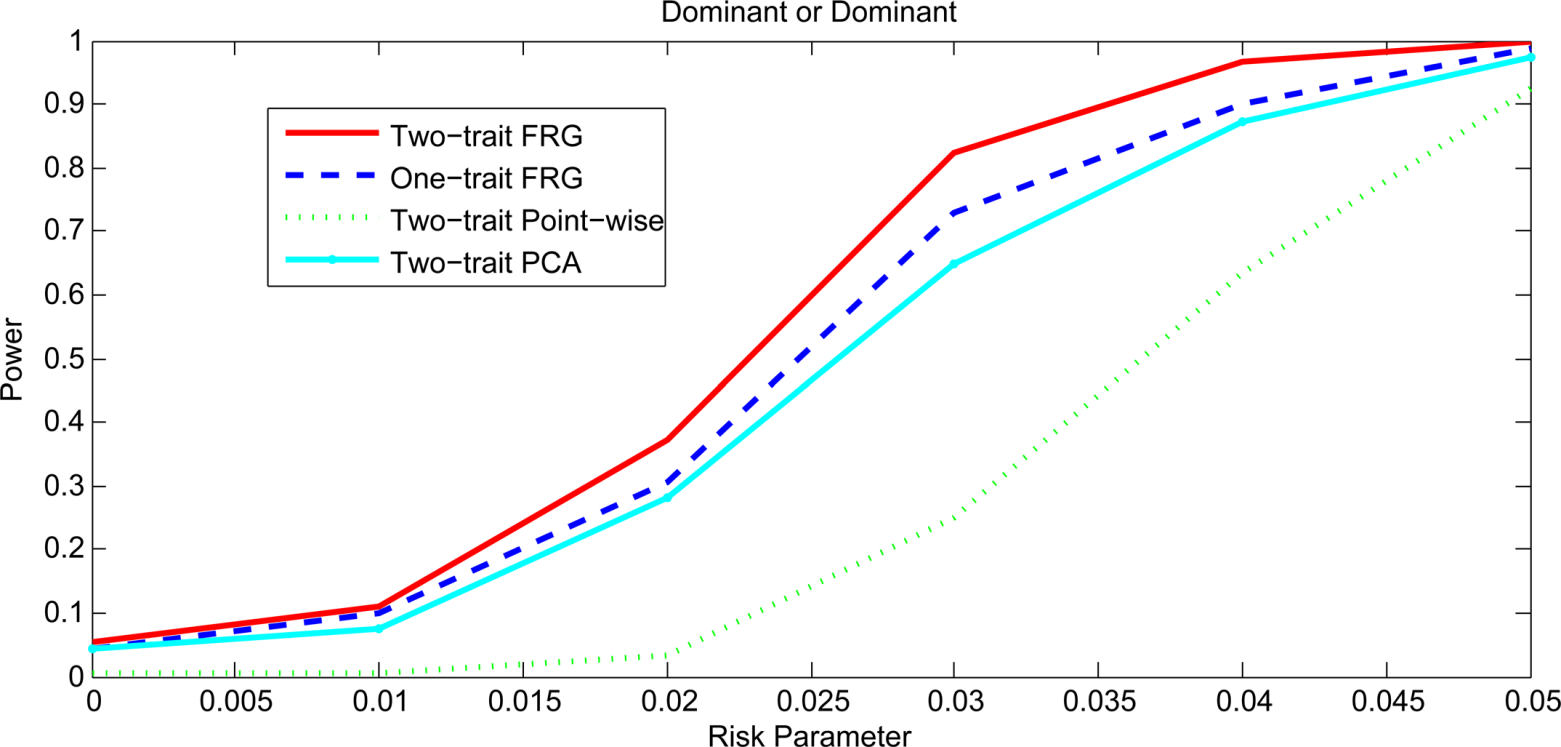
Power curves under Dominant OR Dominant with two genes including common variants only.

**Fig 4:**
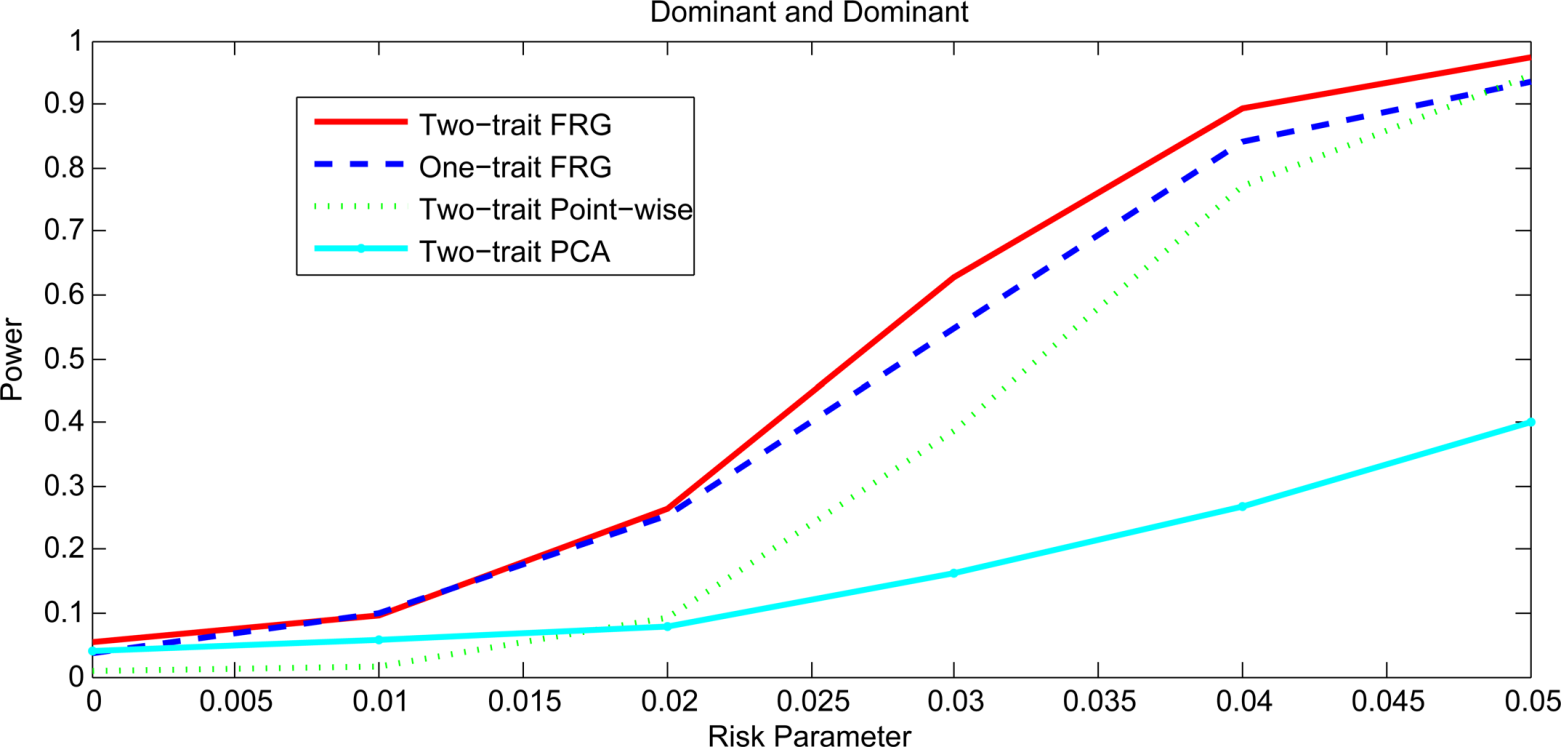
Power curves under Dominant AND Dominant with two genes including common variants only.

Figs 3 and 4, S3 Fig and S4 Fig plotted the power curves of the two-trait FRG, single trait FRG, two-trait regression on PCs and two-trait pair-wise interaction tests for a quantitative trait under Dominant OR Dominant, Dominant AND Dominant, Threshold and Recessive OR Recessive models, respectively. Only two genes include common variants. These power curves are a function of the risk parameter at the significance level α = 0.05. Permutations in the point-wise interaction tests were used to adjust for multiple testing. These figures showed that the power patterns of the epistasis tests for common variants were similar to that for rare variants.

**Fig 5:**
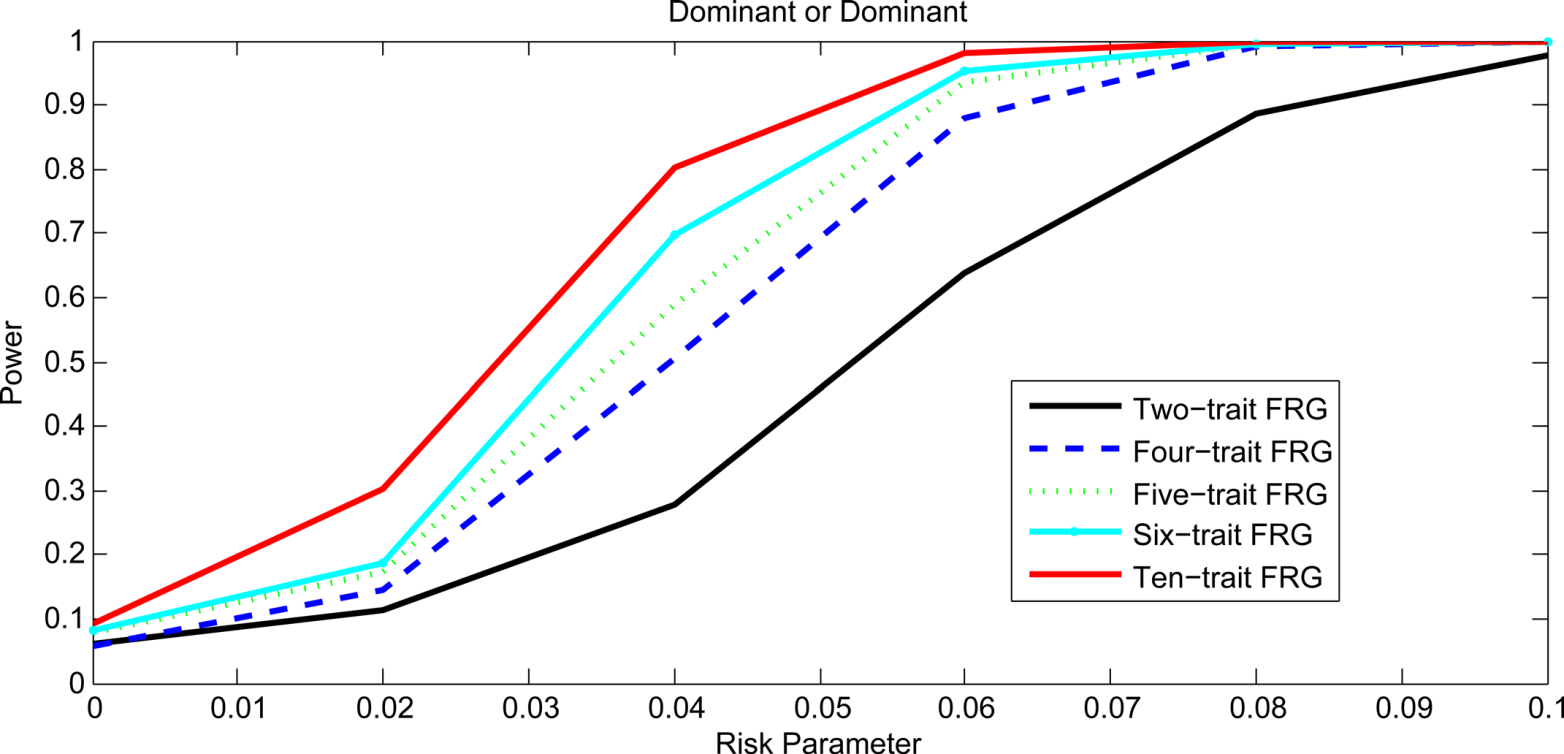
Power curves of MFRG with different trait number under Dominant OR Dominant.

Next we investigate the impact of the number of traits on the power. Fig 5 plotted the power curves of two-trait FRG, four-trait FRG, five-trait FRG, six-trait FRG and ten-trait FRG under Dominant OR Dominant interaction model. Fig 5 showed that if the multiple phenotypes are correlated then the power of the MFRG to detect epistasis will increase as the number of phenotypes increases.

**Fig 6:**
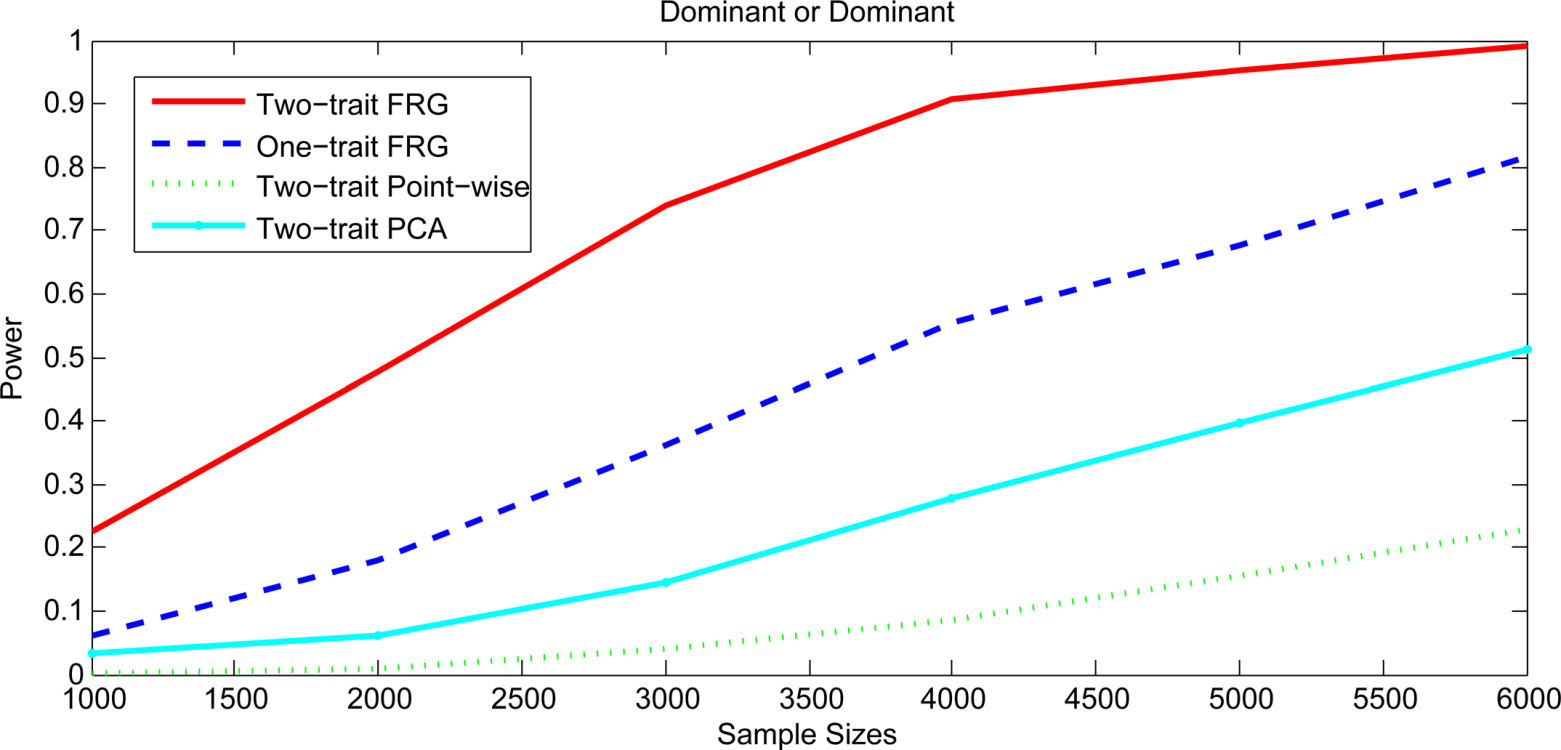
Power curves as a function of sample sizes under Dominant OR Dominant with two genes including rare variants only.

To investigate the impact of sample size on the power, we plotted Fig 6 and S5–S7 Figs showing the power of three statistics for testing the interaction between two genomic regions (or genes) with only rare variants as a function of sample sizes under four interaction models, assuming 20% of the risk rare variants and the risk parameter *r* = 0.05 for Dominant OR Dominant, Dominant AND Dominant, and Recessive OR Recessive, and *r* = 0.5 for Threshold models, respectively. Again, we observed that the power of the two-trait FRG was the highest.

### Application to Real Data Examples

To further evaluate the performance, the MFRG for testing epistasis was applied to data from the NHLBI’s ESP Project. Five phenotypes: HDL, LDL, total cholesterol, SBP and DBP were considered with a total of 2,016 individuals of European origin from 15 different cohorts in the ESP Project. No evidence of cohort- and/or phenotype-specific effects, or other systematic biases was found [34]. Exomes from related individuals were excluded from further analysis. We took the rank-based inverse normal transformation of the phenotypes [35] as trait values. The total number of genes tested for interactions which included both common and rare variants was 18,587. The remaining annotated human genes which did not contain any SNPs in our dataset were excluded from the analysis. A P-value for declaring significant interaction after applying the Bonferroni correction for multiple tests was 2.89×10^−10^. Population stratification may inflate the test statistics. To reduce the inflation, the standard strategy is to adjust for population stratification via principal components. All the tests were adjusted for sex, age and population stratification via 5 principal components.

**Fig 7:**
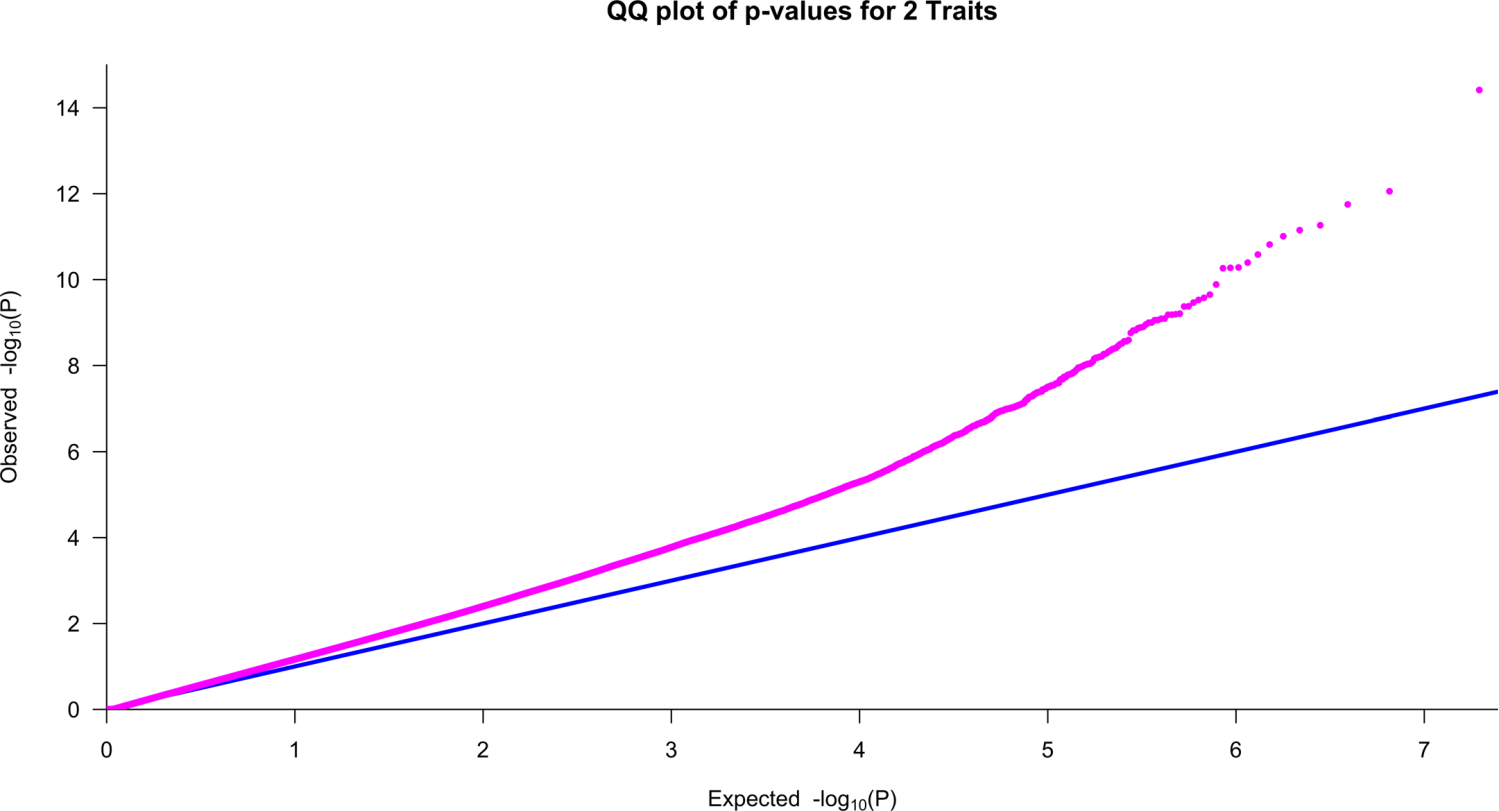
QQ plot of the two-trait FRG test adjusted for sex, age and population stratification via five PCs.

To examine the behavior of the MFRG, we plotted the QQ plot of the two-trait FRG test (Fig 7). The QQ plots showed that the false positive rate of the MFRG for detection of interaction in some degree is controlled.

A total of 91 pairs of genes which were derived from 85 genes showed significant evidence of epistasis with P-values < 2.7×10^−10^ which were calculated using the MFRG model and simultaneously analyzing interaction of inverse normally transformed HDL and LDL (S10 Table). The top 30 pairs of significantly interacted genes with HDL and LDL were listed in Table 5. In Table 5 and S10 Table, P-values for testing interactions between genes by regression on PCA and the minimum of P-values for testing all possible pairs of SNPs between two genes using standard regression model simultaneously analyzed for the HDL and LDL and P-values for testing epistasis by the FRG separately against single trait HDL or LDL were also listed.

**Table 5:** P-values of top 30 pairs of significantly interacted genes with HDL and LDL after adjusting for sex, age and population stratification via five PCs.

Gene 1	Chr	Gene 2	Chr	P-values				
				Two Traits			HDL	LDL
				MFRG	Pair-wise	PCA	FRG	FRG
					(minimum)			
*SHPK*	17	*ST20*	15	1.42E-19	4.30E-04	3.03E-02	2.36E-08	6.48E-11
*STK3*	8	*CSMD1*	8	5.98E-16	5.58E-04	1.76E-01	2.82E-07	6.06E-05
*ST20*	15	*FRMD5*	15	6.97E-15	7.14E-04	2.13E-03	9.36E-07	4.03E-07
*C5orf64*	5	*PSMD1*	2	9.81E-15	1.21E-05	2.64E-01	1.01E-06	3.75E-07
*ST20*	15	*PDE4DIP*	1	3.65E-14	3.42E-06	2.18E-01	1.88E-03	1.95E-08
*SHPK*	17	*CSMD1*	8	2.44E-13	2.37E-04	4.91E-05	1.26E-03	2.93E-04
*C5orf64*	5	*SPRY1*	4	2.64E-13	1.21E-05	2.94E-03	1.12E-06	1.67E-07
*NARG2*	15	*CSMD1*	8	3.77E-13	8.07E-05	4.74E-02	2.24E-03	1.11E-04
*SIGLEC7*	19	*NBPF1*	1	4.03E-13	6.34E-04	2.64E-02	5.04E-06	5.90E-07
*SHPK*	17	*NRG1*	8	5.33E-13	3.91E-04	3.73E-03	2.48E-04	1.58E-07
*PLTP*	20	*NBPF1*	1	8.35E-13	2.03E-03	2.03E-01	6.77E-06	6.34E-05
*DIAPH3-AS1*	13	*SPRY1*	4	1.06E-12	1.35E-05	5.48E-04	1.35E-04	1.34E-06
*MPG*	16	*NBPF1*	1	1.11E-12	3.77E-03	5.56E-01	3.34E-04	2.18E-07
*FRMD5*	15	*SLC8A3*	14	2.92E-12	7.85E-05	9.79E-04	2.99E-03	1.67E-08
*DIAPH3-AS1*	13	*PPRC1*	10	3.57E-12	9.90E-04	7.71E-01	3.33E-05	2.01E-06
*DIAPH3-AS1*	13	*STK3*	8	3.57E-12	3.64E-03	4.74E-01	1.82E-06	5.95E-05
*CD300A*	17	*CSMD1*	8	3.89E-12	3.19E-04	2.59E-01	3.65E-04	1.71E-04
*RNF40*	16	*DIAPH3-AS1*	13	4.09E-12	3.39E-03	2.68E-01	5.05E-05	1.37E-07
*CGB2*	19	*CSMD1*	8	4.38E-12	1.82E-04	3.19E-04	1.87E-05	5.23E-06
*SHPK*	17	*RYR3*	15	4.47E-12	3.25E-04	2.47E-05	1.14E-02	7.29E-05
*FRMD5*	15	*C5orf64*	5	4.75E-12	1.42E-02	3.65E-01	8.14E-06	4.60E-05
*PPM1A*	14	*CSMD1*	8	4.77E-12	6.84E-05	2.37E-03	1.09E-06	2.46E-05
*CSMD1*	8	*ZBTB47*	3	5.53E-12	4.02E-06	2.71E-04	9.85E-07	1.78E-03
*ST20*	15	*PSMD1*	2	5.75E-12	1.06E-05	9.13E-02	4.41E-06	4.93E-06
*CSMD1*	8	*KIF3A*	5	6.56E-12	9.52E-04	2.35E-02	7.04E-03	1.16E-02
*TRIM22*	11	*SORCS2*	4	6.62E-12	1.05E-04	2.01E-01	2.98E-07	1.52E-05
*CREBBP*	16	*CSMD1*	8	7.56E-12	2.64E-05	2.77E-12	2.78E-03	3.16E-03
*ADRA1B*	5	*PSMD1*	2	9.05E-12	1.08E-05	5.86E-01	1.70E-05	1.04E-05
*TRIM22*	11	*STK3*	8	1.17E-11	3.46E-04	5.43E-02	1.48E-06	6.03E-05
*DIAPH3-AS1*	13	*SH2B3*	12	1.25E-11	5.62E-04	3.94E-01	5.91E-06	6.44E-05

Several remarkable features from these results were observed. First, we observed that although pairs of genes showed no strong evidence of interactions influencing individual trait HDL or LDL, they indeed demonstrated significant interactions if interactions were simultaneously analyzed for correlated HDL and LDL. Second, the MFRG often had a much smaller P-value to detect interaction than regression on the PCA and the minimum of P-values of pair-wise tests.

**Fig 8:**
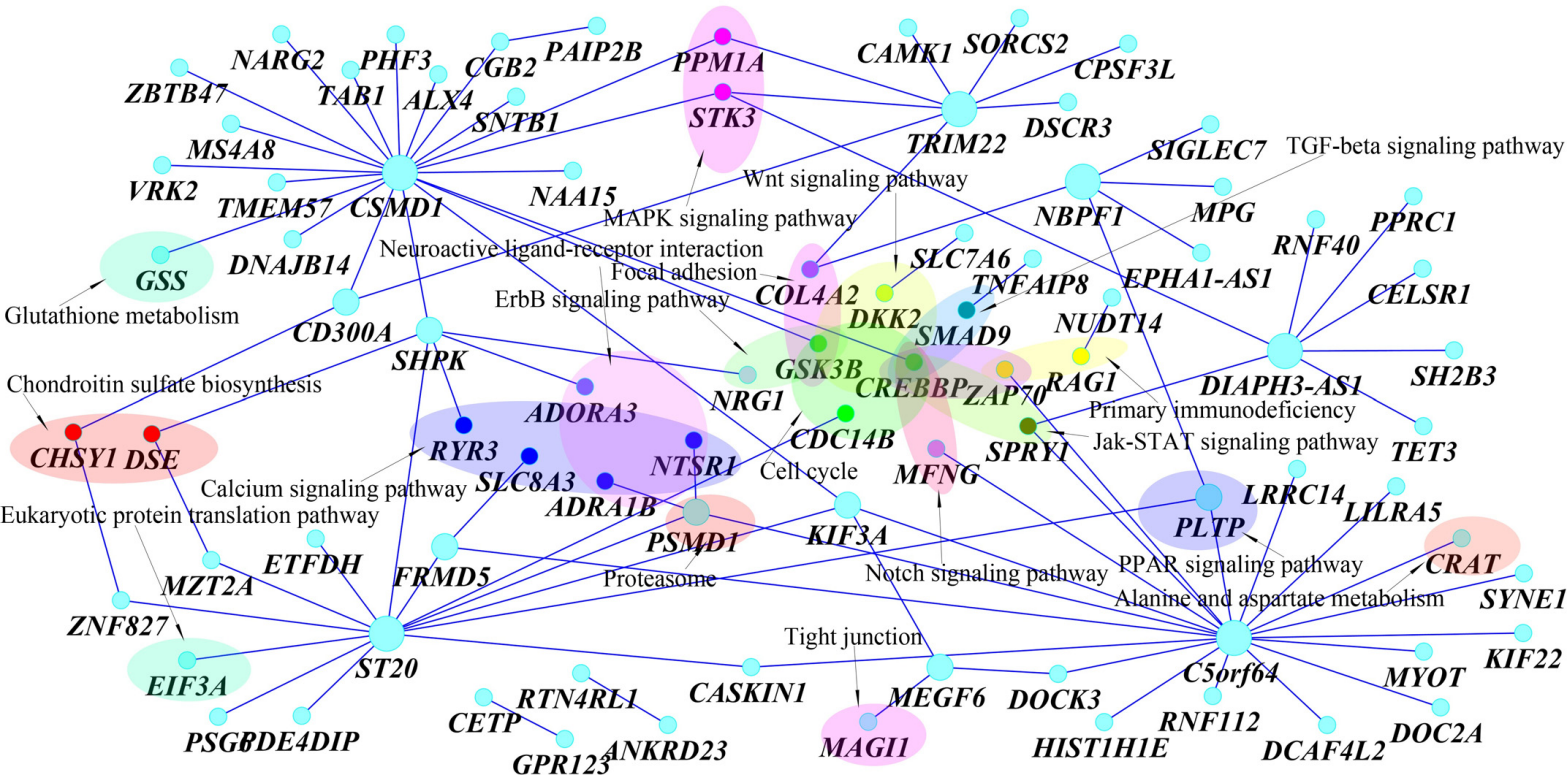
Networks of 91 pairs of genes showing significant evidence of interactions as identified by MFRG.

Third, pairs of SNPs between two genes jointly have significant interaction effects, but individually each pair of SNPs make mild contributions to the interaction effects as shown in Table 6. There were a total of 60 pairs of SNPs between genes *CETP* on chromosome 16 and *GPR123* on chromosome 10 with P-values < 0.0488. None of the 60 pairs of SNPs showed strong evidence of interaction. However, a number of pairs of SNPs between genes *CETP* and *GPR123* collectively demonstrated significant interaction influencing the traits HDL and LDL. Fourth, 91 pairs of interacting genes formed a network (Fig 8). The genes *C5orf64* that had interactions with 19 genes, *CSMD1* that had interactions with 20 genes, were hub genes in the network. 26 genes out of total 85 genes in the network were mainly located in 18 pathways. Each of 12 pathways included at least two interacting genes. However, the majority of interacting genes are located in different pathways. Among 18 pathways, calcium signaling pathway mediates the effect of LDL and plays a role in control of atherosclerosis susceptibility [36], LDL-cholesterol has multiple roles in regulating focal adhesion dynamics [37], LDL is involved in free radical induced apoptosis pathway [38], MAPK and JAK-STAT pathways are involved in dietary flavonoid protection against oxidized LDL [39], up-regulation of autophagy via AMPK/mTOR signaling pathway alleviates oxidized -LDL induced inflammation [40], PPARα holds a fundamental role in control of lipid homeostasis [41] and lectin-like ox-LDL receptor 1 mediates PKC-α/ERK/PPAR-γ/MMP pathway [42], HDL reduces the TGF-β1-induced collagen deposition [43], the Wnt pathway plays an important role in lipid storage and homeostasis [44], From the literatures, we found that both common and rare variants in *CETP* were associated with the HDL [45], *CREBBP* regulated LDL receptor transcription [46], *PLTP* was associated with HDL and LDL [47], *TMEM57* was associated with serum lipid levels [48], *SH2B3* was associated with LDL cholesterol [49]. It was also reported that *CSMD1* was associated with multivariate phenotype defined as low levels of low density lipoprotein cholesterol (LDL-C < or = 100 mg/dl) and high levels of triglycerides (TG > or = 180 mg/dl) [50], associated with hypertension [51]. It was also reported that *CSMD1* was associated with LDL and total cholesterol [52].

**Table 6:** P-values of 60 pairs of SNPs between genes CETP on chromosome 16 and GPR123 on chromosome 10 for testing interaction affecting both HDL and LDL.

Gene 1			Gene 2			P-value
*CETP*			*GPR123*			8.83E-11
SNP1	BP	MAF	SNP2	BP	MAF	P-Value
rs9930761	57007192	0.0672123	rs367825198	134940686	0.00024802	4.53E-05
rs5883	57007353	0.0577877	rs367825198	134940686	0.00024802	4.90E-05
rs148628525	56995963	0.00024802	rs11101914	134910629	0.25198413	1.14E-03
rs1800777	57017319	0.03497024	rs2806452	134942166	0.36383929	2.58E-03
rs1800774	57015545	0.34945437	rs115735367	134940724	0.0014881	3.03E-03
rs140547417	57009022	0.00124008	rs2806452	134942166	0.36383929	3.46E-03
rs5883	57007353	0.0577877	rs12219529	134916366	0.14409722	3.69E-03
rs140547417	57009022	0.00124008	rs11101914	134910629	0.25198413	3.94E-03
rs5883	57007353	0.0577877	rs2806453	134942319	0.04464286	5.22E-03
rs9930761	57007192	0.0672123	rs115735367	134940724	0.0014881	5.98E-03
rs5883	57007353	0.0577877	rs2806452	134942166	0.36383929	6.07E-03
rs1800777	57017319	0.03497024	rs10776696	134942340	0.11433532	6.12E-03
rs1532625	57005301	0.41914683	rs115735367	134940724	0.0014881	7.16E-03
rs9930761	57007192	0.0672123	rs118125186	134912135	0.00421627	7.23E-03
rs5883	57007353	0.0577877	rs118125186	134912135	0.00421627	7.50E-03
rs1532625	57005301	0.41914683	rs145543174	134941843	0.00049603	7.90E-03
rs1532625	57005301	0.41914683	rs118125186	134912135	0.00421627	8.49E-03
rs140547417	57009022	0.00124008	rs45586231	134942832	0.06547619	8.93E-03
rs9930761	57007192	0.0672123	rs12219529	134916366	0.14409722	9.17E-03
rs1532625	57005301	0.41914683	rs45586231	134942832	0.06547619	9.23E-03
rs34065661	56995935	0.00124008	rs115735367	134940724	0.0014881	9.56E-03
rs140547417	57009022	0.00124008	rs11101942	134940862	0.11929563	9.62E-03
rs9930761	57007192	0.0672123	rs2806452	134942166	0.36383929	9.69E-03
rs140547417	57009022	0.00124008	rs10776696	134942340	0.11433532	9.76E-03
rs1532625	57005301	0.41914683	rs4838796	134912098	0.03298611	1.12E-02
rs1800774	57015545	0.34945437	rs118125186	134912135	0.00421627	1.19E-02
rs1800774	57015545	0.34945437	rs2806452	134942166	0.36383929	1.33E-02
rs5883	57007353	0.0577877	rs11101916	134912314	0.1703869	1.37E-02
rs5883	57007353	0.0577877	rs11101942	134940862	0.11929563	1.46E-02
rs5883	57007353	0.0577877	rs45586231	134942832	0.06547619	1.59E-02
rs371233223	57005272	0.00024802	rs11101916	134912314	0.1703869	1.68E-02
rs371233223	57005272	0.00024802	rs2806452	134942166	0.36383929	1.70E-02
rs1532625	57005301	0.41914683	rs2806452	134942166	0.36383929	1.75E-02
rs13306230	57003250	0.00124008	rs2806452	134942166	0.36383929	2.21E-02
rs34611098	57004951	0.00124008	rs2806452	134942166	0.36383929	2.21E-02
rs5880	57015091	0.04861111	rs2806452	134942166	0.36383929	2.23E-02
rs9930761	57007192	0.0672123	rs11101941	134940779	0.01116071	2.28E-02
rs1800777	57017319	0.03497024	rs118125186	134912135	0.00421627	2.33E-02
rs5880	57015091	0.04861111	rs118125186	134912135	0.00421627	2.35E-02
rs9930761	57007192	0.0672123	rs2806453	134942319	0.04464286	2.47E-02
rs182237338	57012174	0.00198413	rs12219529	134916366	0.14409722	2.57E-02
rs5883	57007353	0.0577877	rs11101941	134940779	0.01116071	2.68E-02
rs5880	57015091	0.04861111	rs11101916	134912314	0.1703869	2.94E-02
rs9930761	57007192	0.0672123	rs45586231	134942832	0.06547619	2.95E-02
rs9930761	57007192	0.0672123	rs11101914	134910629	0.25198413	3.19E-02
rs376545293	57016085	0.00024802	rs11101916	134912314	0.1703869	3.23E-02
rs9930761	57007192	0.0672123	rs11101942	134940862	0.11929563	3.24E-02
rs376545293	57016085	0.00024802	rs11101914	134910629	0.25198413	3.28E-02
rs139594305	57007286	0.00024802	rs4838796	134912098	0.03298611	3.46E-02
rs376545293	57016085	0.00024802	rs2806452	134942166	0.36383929	3.48E-02
rs139594305	57007286	0.00024802	rs12219529	134916366	0.14409722	3.55E-02
rs201267603	57005220	0.00099206	rs12219529	134916366	0.14409722	3.85E-02
rs13306230	57003250	0.00124008	rs11101916	134912314	0.1703869	4.00E-02
rs34611098	57004951	0.00124008	rs11101916	134912314	0.1703869	4.00E-02
rs9930761	57007192	0.0672123	rs11101916	134912314	0.1703869	4.11E-02
rs1800774	57015545	0.34945437	rs189113844	134941821	0.00173611	4.35E-02
rs1800774	57015545	0.34945437	rs11101941	134940779	0.01116071	4.38E-02
rs28381708	57007413	0.00124008	rs11101914	134910629	0.25198413	4.57E-02
rs34855278	57015076	0.0014881	rs11101914	134910629	0.25198413	4.69E-02
rs1532625	57005301	0.41914683	rs10776696	134942340	0.11433532	4.88E-02

Next we analyzed five traits: HDL, LDL, SBP, DBP and TOTCHOL. Again, for each trait, inverse normal rank transformation was conducted to ensure that the normality assumption of the transformed trait variable was valid. To examine the behavior of the MFRG, we plotted QQ plot of the test (S8 Fig). The QQ plots showed that the false positive rate of the MFRG for detection of interaction is controlled.

**Fig 9:**
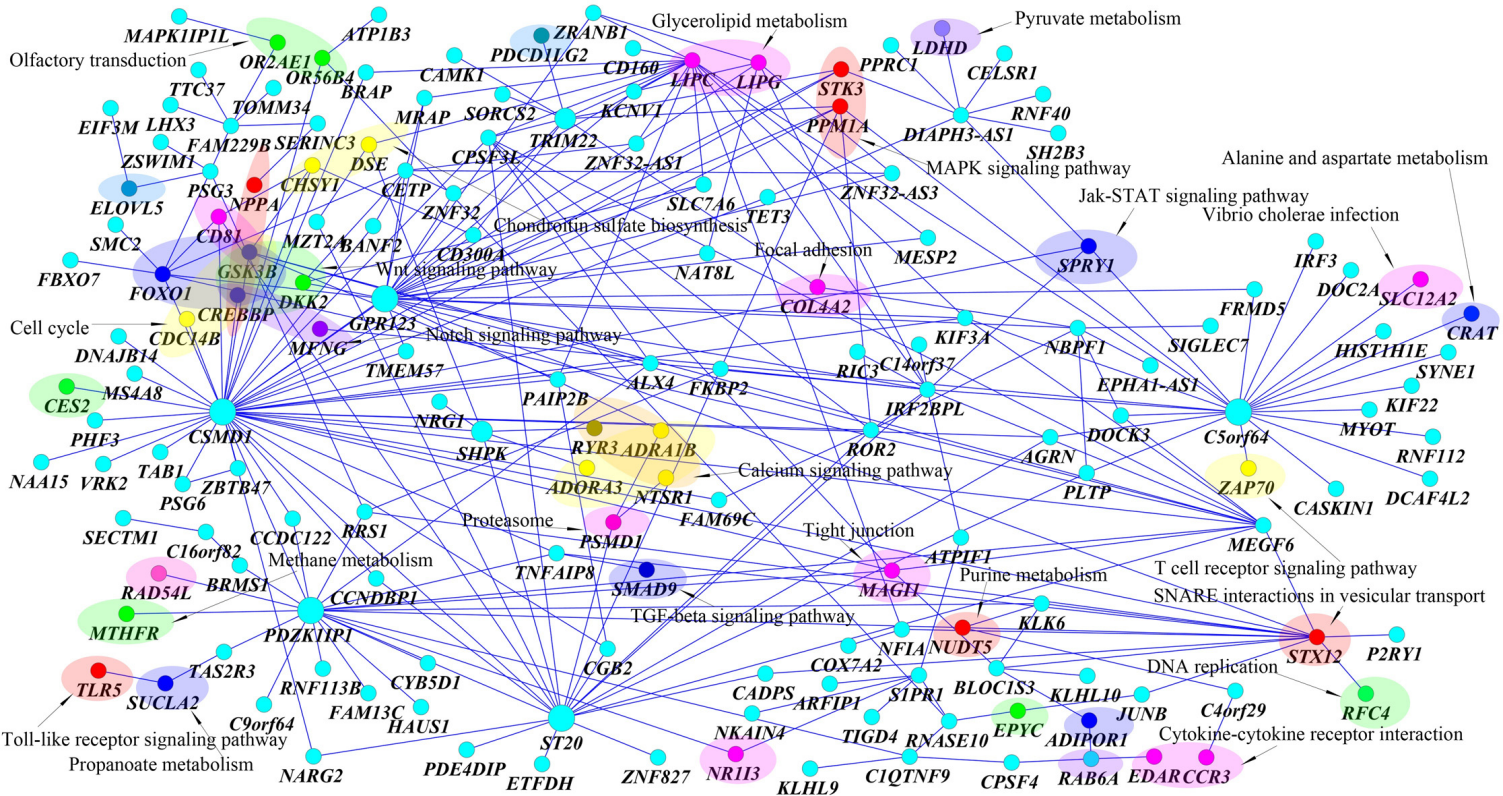
Networks of 267 pairs of genes showing significant evidence of interactions as identified by MFRG.

A total of 267 pairs of genes which were derived from 160 genes showed significant evidence of epistasis influencing five traits with P-values < 1.96×10^−10^ which were calculated using the MFRG model (S11 Table). Of them formed a largest connected subnetwork (Fig 9). The top 25 pairs of significantly interacted genes with five traits were listed in Table 7. We observed the same pattern as was observed for the two traits: HDL and LDL. 46 genes out of 160 genes in the networks were mainly located in 42 pathways including 15 signaling pathways. Among them, 14 pathways were in Fig 8. The interacting genes may be involved in the same biological pathway or in the different biological pathways. We observed 12 pathways, each of which contained at least two genes connected via interaction. However, the majority of interacting genes were not located in the same pathways.

**Table 7:** P-values of top 25 pairs of significantly interacted genes with five traits.

Gene 1	Gene 2	P-values							
		Five Traits			LDL	HDL	SBP	DBP	TOTCHOL
		MFRG	Pair-wise(min)	PCA	FRG	FRG	FRG	FRG	FRG
*PDZK1IP1*	*CSMD1*	4.29E-35	5.61E-16	1.36E-10	4.52E-03	6.44E-02	8.62E-03	2.43E-03	1.19E-03
*STK3*	*CSMD1*	5.84E-34	3.39E-05	5.81E-03	6.06E-05	2.82E-07	1.37E-05	1.79E-05	3.22E-04
*MEGF6*	*IRF2BPL*	1.51E-31	2.55E-23	3.33E-18	7.65E-01	8.99E-02	2.75E-03	4.07E-02	1.25E-01
*PLTP*	*C5orf64*	1.52E-31	2.96E-03	9.32E-01	1.77E-04	3.39E-07	3.37E-08	1.61E-06	3.26E-04
*CSMD1*	*CCNDBP1*	3.57E-31	1.66E-05	9.71E-04	1.14E-04	3.57E-03	8.80E-05	4.84E-04	8.23E-04
*KIF3A*	*C5orf64*	1.06E-30	4.94E-05	5.63E-02	7.44E-05	1.21E-06	2.03E-03	1.50E-03	1.05E-03
*CSMD1*	*KIF3A*	1.10E-30	3.97E-05	1.13E-05	1.16E-02	7.04E-03	3.60E-01	3.54E-01	1.42E-02
*ST20*	*PDE4DIP*	1.93E-30	1.14E-06	6.03E-02	1.95E-08	1.88E-03	2.06E-03	3.22E-03	2.11E-06
*CSMD1*	*NARG2*	2.86E-30	3.97E-05	1.90E-06	1.11E-04	2.24E-03	1.78E-02	1.90E-03	3.56E-04
*PDZK1IP1*	*ST20*	3.17E-30	9.82E-16	2.09E-03	6.15E-03	1.34E-02	2.30E-03	7.63E-03	3.30E-03
*CSMD1*	*FOXO1*	3.32E-30	3.01E-19	2.59E-08	1.49E-06	2.61E-06	1.49E-05	9.21E-07	8.65E-07
*SHPK*	*ST20*	1.11E-29	3.27E-03	9.68E-02	6.48E-11	2.36E-08	4.26E-04	8.34E-05	1.63E-09
*DIAPH3-AS1*	*SPRY1*	2.65E-29	5.40E-08	2.07E-06	1.34E-06	1.35E-04	2.47E-02	8.94E-02	7.67E-05
*PLTP*	*NBPF1*	4.60E-29	2.92E-03	4.62E-01	6.34E-05	6.77E-06	2.85E-04	8.16E-03	3.93E-05
*CREBBP*	*CSMD1*	2.18E-28	2.34E-05	5.30E-26	3.16E-03	2.78E-03	1.33E-01	6.30E-02	2.21E-03
*TAB1*	*CSMD1*	2.21E-28	1.33E-03	1.34E-04	5.12E-02	2.51E-02	1.48E-02	6.86E-02	4.17E-02
*PAIP2B*	*CSMD1*	2.79E-28	2.54E-07	3.24E-12	1.02E-06	3.85E-06	1.24E-08	8.12E-08	1.38E-05
*CSMD1*	*PHF3*	2.95E-28	1.31E-04	7.20E-09	3.61E-03	6.28E-02	9.38E-02	6.87E-02	1.82E-03
*CCNDBP1*	*ST20*	3.19E-28	3.57E-03	1.32E-03	2.08E-05	1.32E-07	2.77E-06	1.17E-06	2.30E-05
*SHPK*	*CSMD1*	8.06E-28	1.90E-03	1.77E-05	2.93E-04	1.26E-03	2.88E-02	2.68E-02	4.14E-04
*ROR2*	*IRF2BPL*	9.44E-28	2.02E-23	2.71E-17	5.04E-02	1.17E-01	2.30E-01	2.20E-01	9.30E-03
*NARG2*	*ST20*	1.25E-27	4.69E-05	1.07E-02	1.00E-04	2.46E-04	6.28E-05	1.28E-05	3.14E-05
*TRIM22*	*CPSF3L*	1.96E-27	1.03E-18	4.10E-05	2.39E-05	9.32E-05	5.34E-05	2.50E-05	3.09E-05
*ST20*	*PSMD1*	5.40E-27	6.32E-08	2.73E-02	4.93E-06	4.41E-06	2.31E-03	1.66E-05	1.24E-05
*KIF3A*	*MEGF6*	7.65E-27	4.46E-06	6.84E-03	3.22E-04	1.07E-04	3.68E-02	7.89E-02	6.58E-04

Again, we observed that pairs of SNPs between two genes jointly have significant interaction effects, but individually each pair of SNPs might make mild contributions to the interaction effects as shown in S12 Table. There were a total of 6,766 pairs of SNPs between genes *CSMD1* and *FOXO1*. S12 Table listed 101 pairs of SNPs with P-values < 0.049. The majority of the 101 pairs of SNPs showed no strong evidence of interaction. However, they collectively demonstrated significant interaction influencing five traits.

Among 42 pathways, in the previous sections we reported that 14 pathways were associated with HDL and LDL. From the literatures, we also know that unsaturated fatty acids stimulated the uptake of the LDL particles [53], PPAR signaling pathway was correlated with blood pressure [54], purine metabolism was associated with SBP [55], Wnt signaling pathway mediated cholesterol transportation [56], glycerolipid metabolism pathway was correlated with total cholesterol [57], focal adhesion pathway was involved in lipid modulation [58], Cell adhesion molecules was correlated with blood pressure [59].

We also observed from the literatures that a number of genes that appeared in the list of interacted genes with five traits had major genetic effects with single trait. Many reports showed that *CETP*, *LIPC* and *LIPG* were associated with HDL and LDL [60–62] and that *MTHRR* had known main effects for LDL [63] and blood pressure [64], *NR1I3* for lipid metabolism [65], *PLTP* for LDL [66],[67], *FOXO1* for LDL [68] and hypertension [69], *SMAD9* for hypertension [70], and *CSMD1* for SBP [51].

## Discussion

Most genetic analyses of phenotypes have focused on analyzing single traits or, analyzing each phenotype independently. However, multiple phenotypes are highly correlated. Genetic variants can be associated with more than one trait. Genetic pleiotropic effects likely play a crucial role in the molecular basis of correlated phenotypes. To address these central themes and critical barriers in interaction analysis of multiple phenotypes, we shift the paradigm of interaction analysis from individual interaction analysis to pleiotropic interaction analysis and uncover the global organization of biological systems. MFRG was used to develop a novel statistical framework for joint interaction analysis of multiple correlated phenotypes. By large simulations and real data analysis the merits and limitations of the proposed new paradigm of joint interaction analysis of multiple phenotypes were demonstrated.

The new approach fully uses all phenotype correlation information to jointly analyze interaction of multiple phenotypes. By large simulations and real data analysis, we showed that the proposed MFRG for joint interaction analysis of correlated multiple phenotypes substantially increased the power to detect interaction while keeping the Type 1 error rates of the test statistics under control. In real data analysis, we observed that although pairs of genes showed no strong evidence of interactions influencing individual trait, they indeed demonstrated significant interactions if interactions were simultaneously analyzed for correlated multiple traits.

Due to lack of power of the widely used statistics for testing interaction between loci and its computational intensity, exploration of genome-wide gene-gene interaction has been limited. Few significant interaction results have been observed. Many geneticists question the universe presence of significant gene-gene interaction. Our analysis showed that although the number of significantly interacted genes for single phenotype was small, the number of significantly interacted genes for multiple phenotypes substantially increased. Our results suggested that joint interaction analysis of multiple phenotypes should be advocated in future genetic studies of complex traits.

The interaction analysis for multiple phenotypes has been limited to common variants in carefully controlled experimental crosses and has mainly focused on the pair-wise interaction analysis. Although pair-wise interaction analysis is suitable for common variants, it is difficult to use to test interaction between rare and rare variants, and rare and common variants. There is an increasing need to develop statistics that can be used to test interactions among the entire allelic spectrum of variants for joint interaction analysis of multiple phenotypes. The MFRG utilizes the merits of taking genotype as functions and decomposes position varying genotype function into orthogonal eigenfunctions of genomic position. Only a few eigenfunctions that capture major information on genetic variation across the gene, are used to model the genetic variation. This substantially reduces the dimension in genetic variation of the data. The MFRG can efficiently test the interaction between rare and rare, rare and common, and common and common variants.

In both real data analysis of two phenotypes and five phenotypes, the interacted genes formed interaction networks. Hub genes in the interaction networks were also observed. These hub genes usually play an important biological role in causing phenotype variation.

An essential issue for interaction analysis of a large number of phenotypes is how to reduce dimension while fully exploiting complementary information in multiple phenotypes. The standard multivariate regression models for joint interaction analysis of multiple phenotypes do not explore the correlation structures of multiple phenotypes and reduce the dimensions of the phenotypes, and hence have limited power to detect pleotropic interaction effects due to large degrees of freedom. Data reduction techniques such as principal component analysis should be explored in the future interaction analysis of multiple phenotypes.

The results in this paper are preliminary. The current marginal approaches for interaction analysis cannot distinguish between direct and indirect interactions, which will decrease our power to unravel mechanisms underlying complex traits. To overcome these limitations, causal inference tools should be explored for the joint interaction analysis of multiple phenotypes. The purpose of this paper is to stimulate further discussions regarding great challenges we are facing in the interaction analysis of high dimensional phenotypic and genomic data produced by modern sensors and next-generation sequencing.

## Supporting Information

S1 Fig Power curves under Threshold with two genes including rare variants only. (TIF)

S2 Fig Power curves under Recessive OR Recessive with two genes including rare variants only. (TIF)

S3 Fig Power curves under Threshold with two genes including common variants only. (TIF)

S4 Fig Power curves under Recessive OR Recessive with two genes including common variants only. (TIF)

S5 Fig Power curves of MFRG with different sample size under Dominant AND Dominant. (TIF)

S6 Fig Power curves of MFRG with different sample size under Recessive OR Recessive. (TIF)

S7 Fig Power curves of MFRG with different sample size under Threshold. (TIF)

S8 Fig QQ plot of the five-trait FRG test for the ESP dataset. (TIF)

S1 Table Average type 1 error rates of the statistic for testing interaction between two genes with no marginal effect consisting of only rare variants with 2 traits over 10 pairs of genes. (DOCX)

S2 Table Average type 1 error rates of the statistic for testing interaction between two genes with no marginal effect consisting of only rare variants with 10 traits over 10 pairs of genes. (DOCX)

S3 Table Average type 1 error rates of the statistic for testing interaction between two genes with marginal effect consisting of only rare variants with 2 traits over 10 pairs of genes. (DOCX)

S4 Table Average type 1 error rates of the statistic for testing interaction between two genes with marginal effect at one gene consisting of only rare variants with 10 traits over 10 pairs of genes. (DOCX)

S5 Table Average type 1 error rates of the statistic for testing interaction between two genes with marginal effects at two genes consisting of only rare variants with 2 traits over 10 pairs of genes. (DOCX)

S6 Table Average type 1 error rates of the statistic for testing interaction between two genes with marginal effects at two genes consisting of only rare variants with 10 traits over 10 pairs of genes. (DOCX)

S7 Table Average type 1 error rates of the statistic for testing interaction between two genes with marginal effects at two genes consisting of only common variants with 2 traits over 10 pairs of genes. (DOCX)

S8 Table Average type 1 error rates of the statistic for testing interaction between two genes with marginal effects at two genes consisting of only common variants with 10 traits over 10 pairs of genes. (DOCX)

S9 Table The interaction models: 0 and r stand for a quantitative trait mean given the genotypes. (DOCX)

S10 Table P-values of significantly interacted genes with HDL and LDL. (XLSX)

S11 Table P-values of significantly interacted genes with HDL, LDL, SBP, DBP and TOTCHOL. (XLSX)

S12 Table P-values of 101 pairs of SNPs between genes CSMD1 and FOXO1 for testing interaction affecting five traits. (XLSX)

S1 Text Appendix: Estimation of interaction effects. (DOCX)
