# Supplementary figures and images for: Functional Regression Models for Epistasis Analysis of Multiple Quantitative Traits
Source: PLoS Genet. 2016 Apr 22;12(4):e1005965. doi: 10.1371/journal.pgen.1005965 (PMC4841563; doi:10.1371/journal.pgen.1005965)

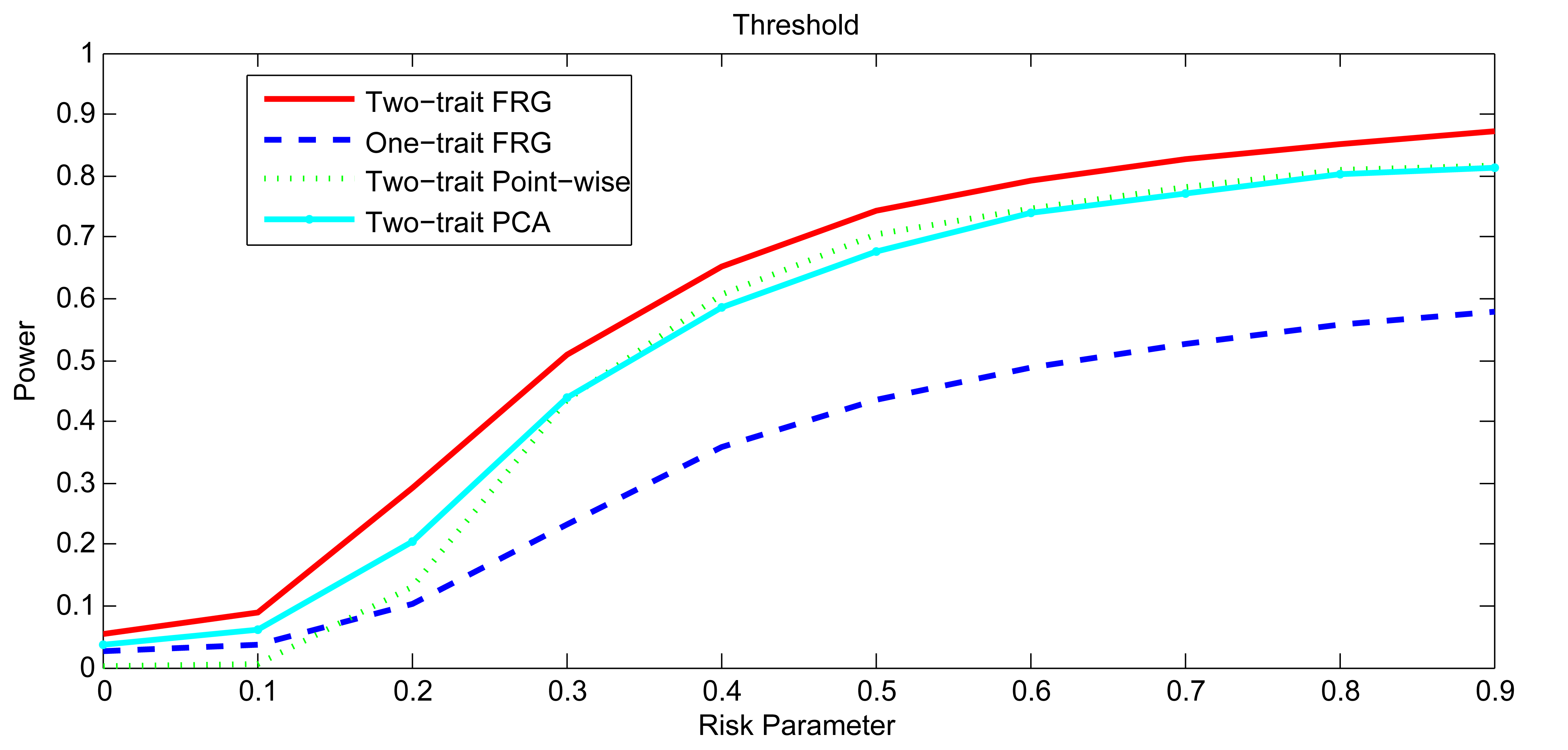

Supplement: S1 Fig — (TIF) [file pgen.1005965.s001.TIF]

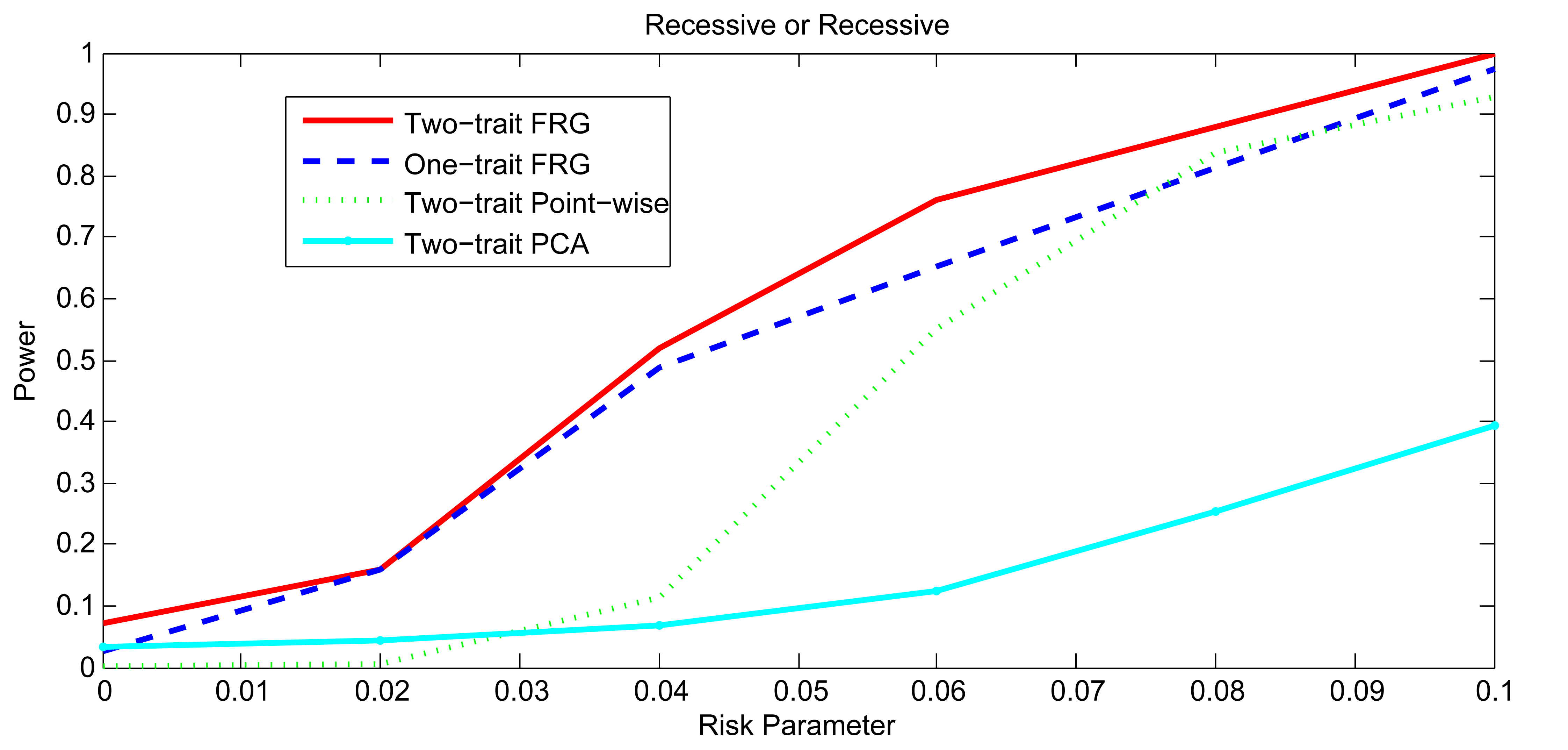

Supplement: S2 Fig — (TIF) [file pgen.1005965.s002.TIF]

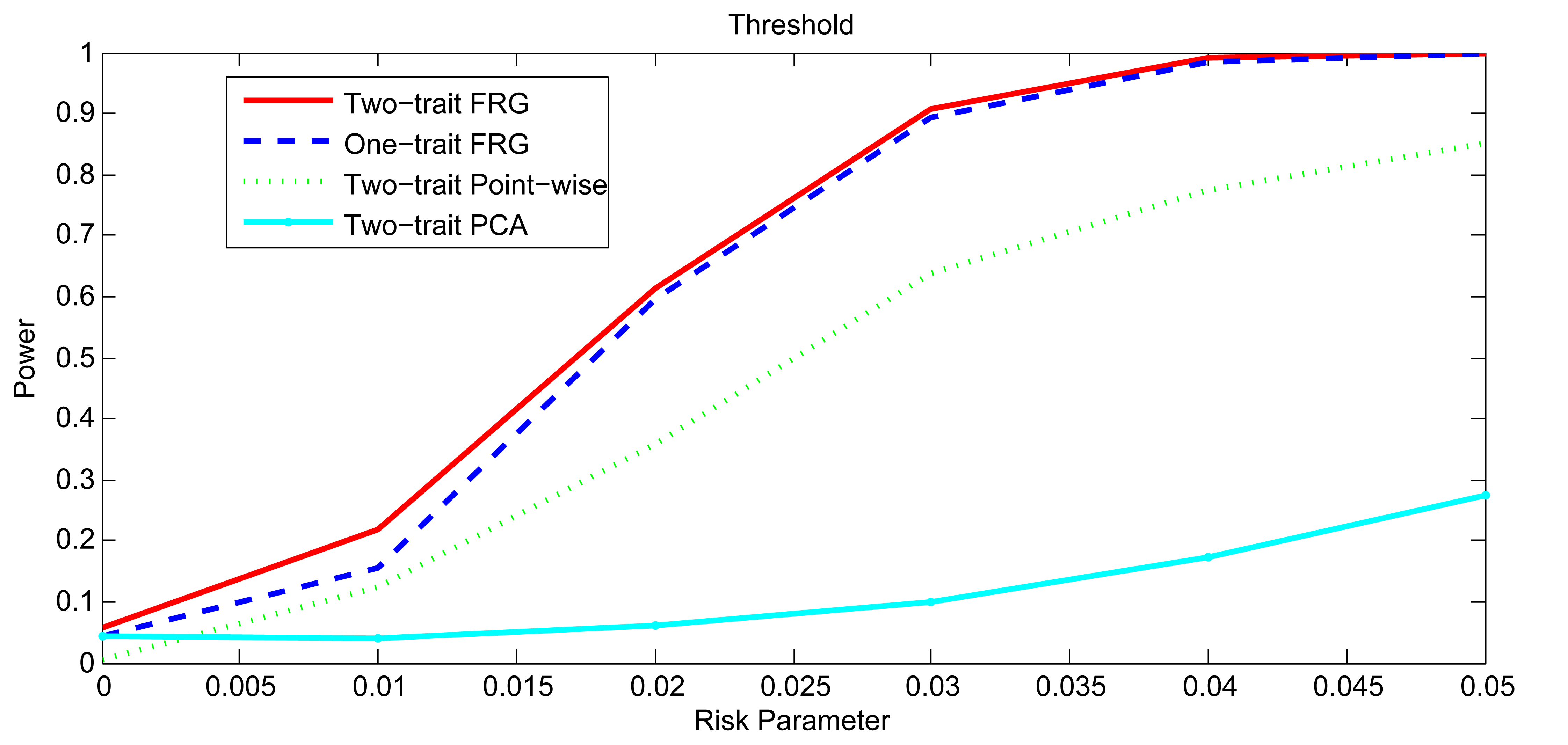

Supplement: S3 Fig — (TIF) [file pgen.1005965.s003.TIF]

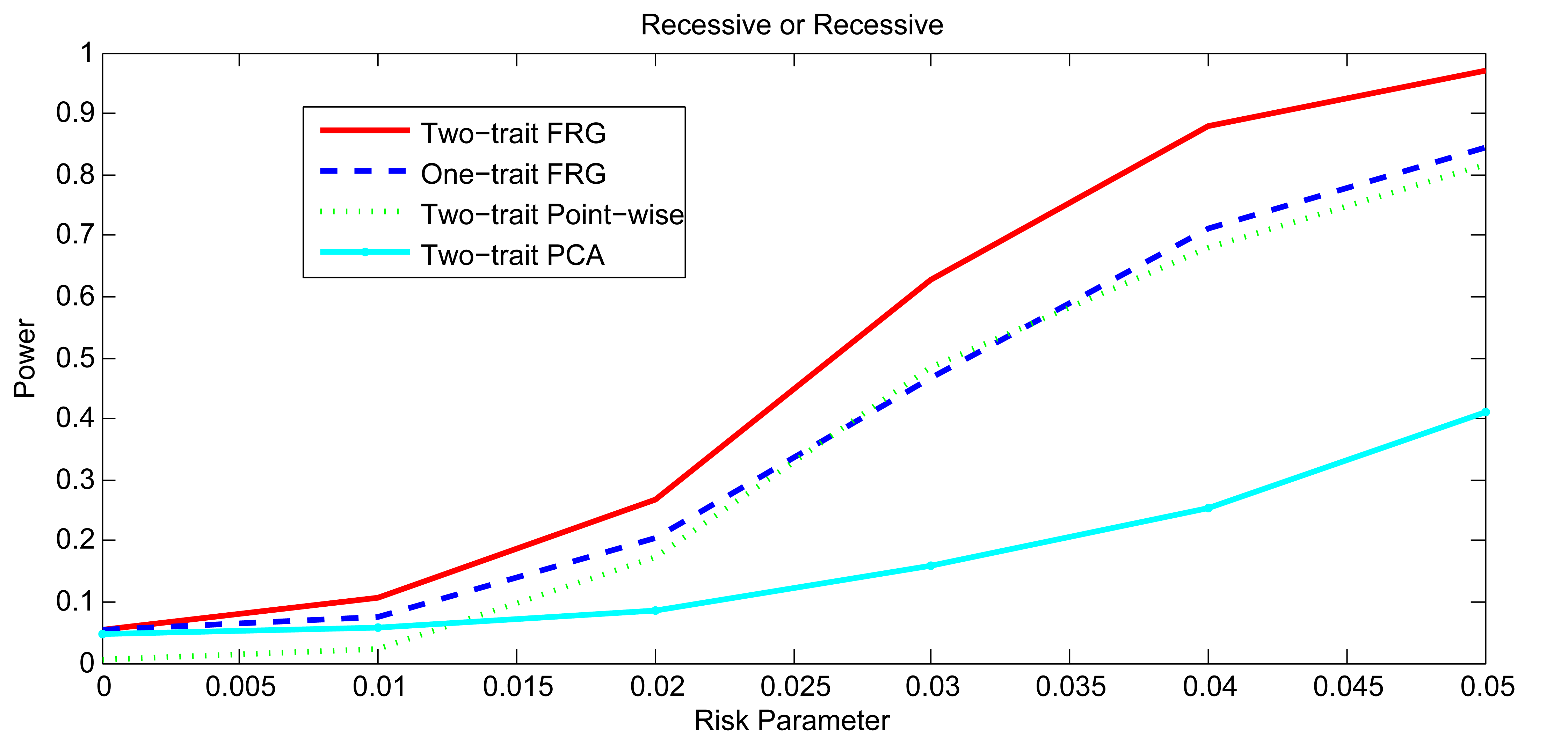

Supplement: S4 Fig — (TIF) [file pgen.1005965.s004.TIF]

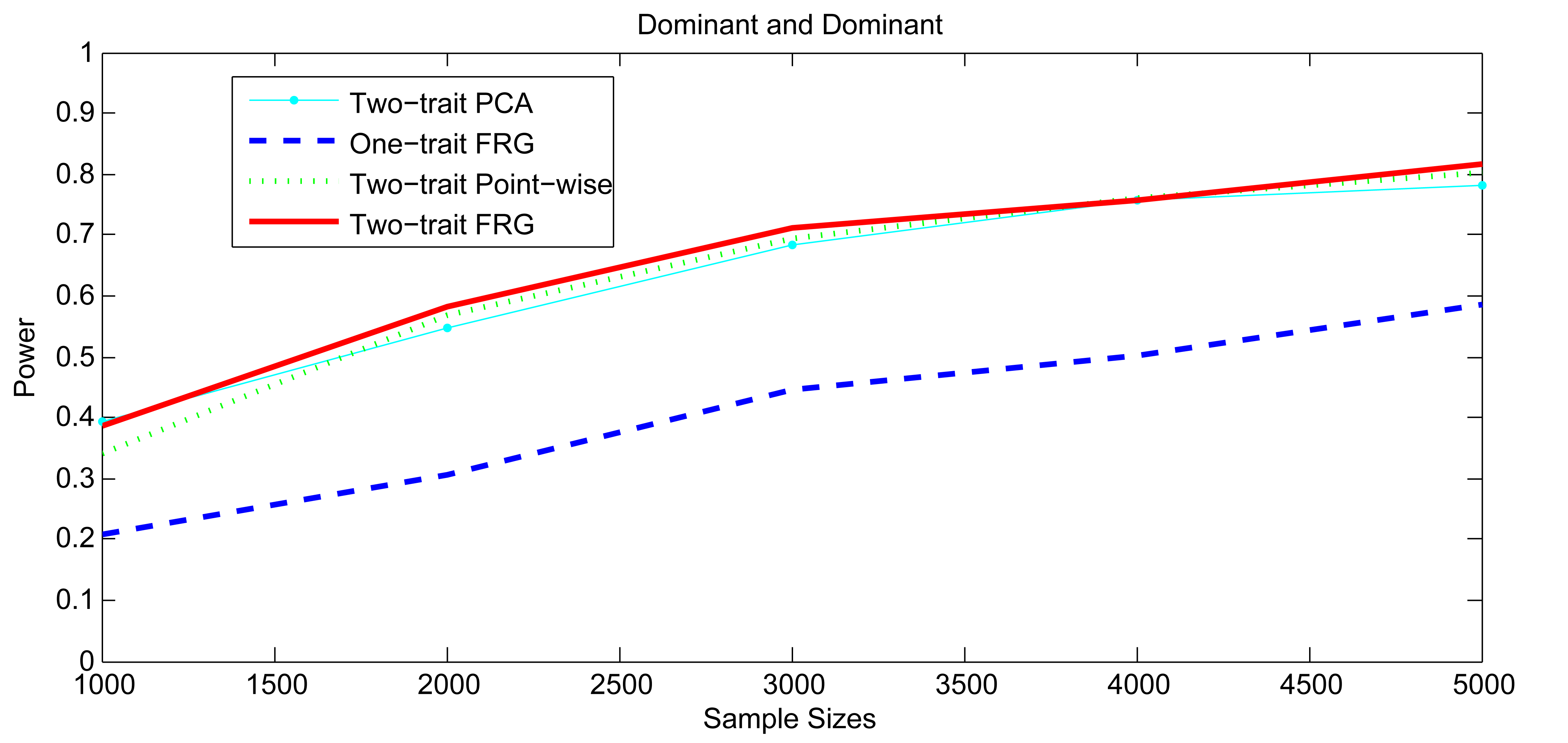

Supplement: S5 Fig — (TIF) [file pgen.1005965.s005.TIF]

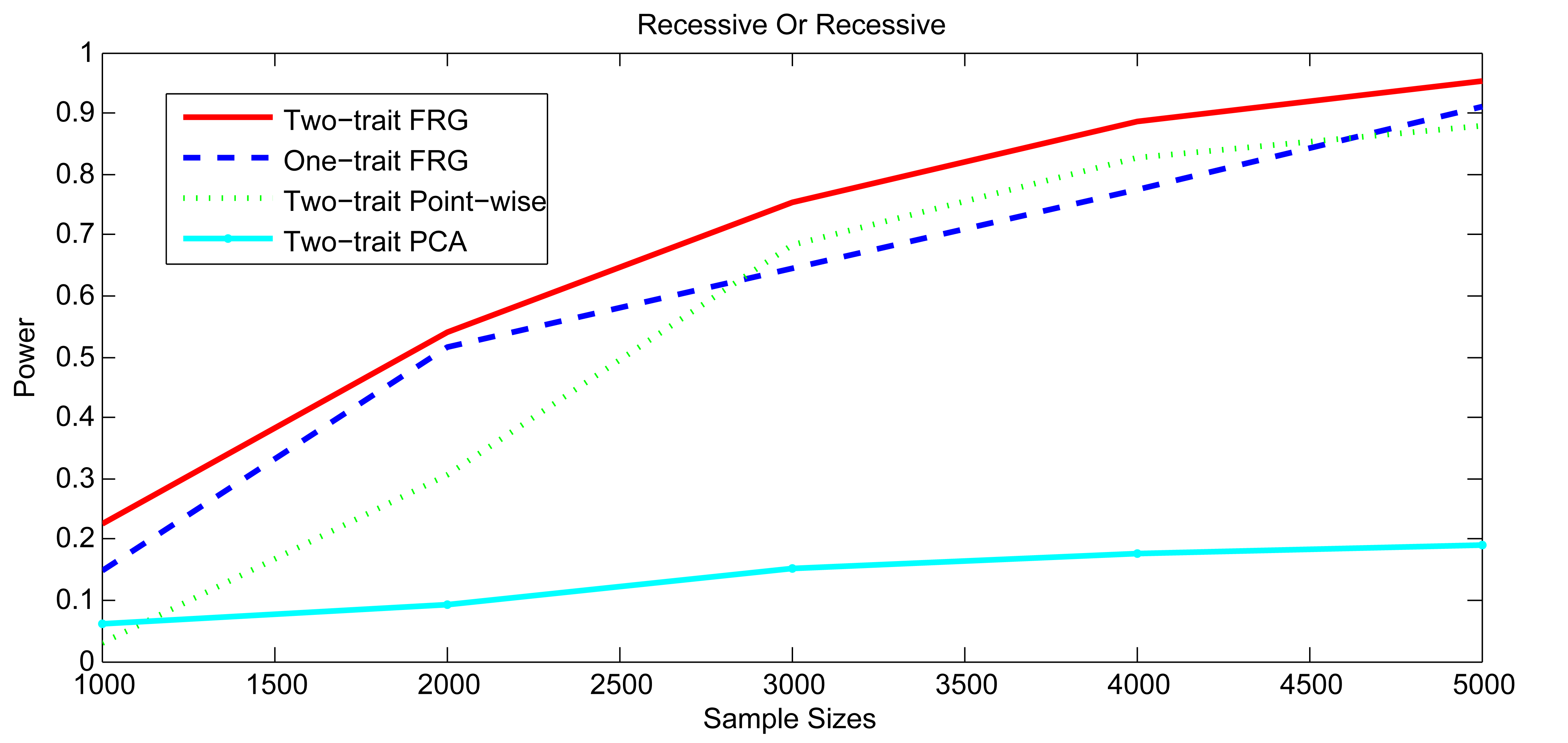

Supplement: S6 Fig — (TIF) [file pgen.1005965.s006.TIF]

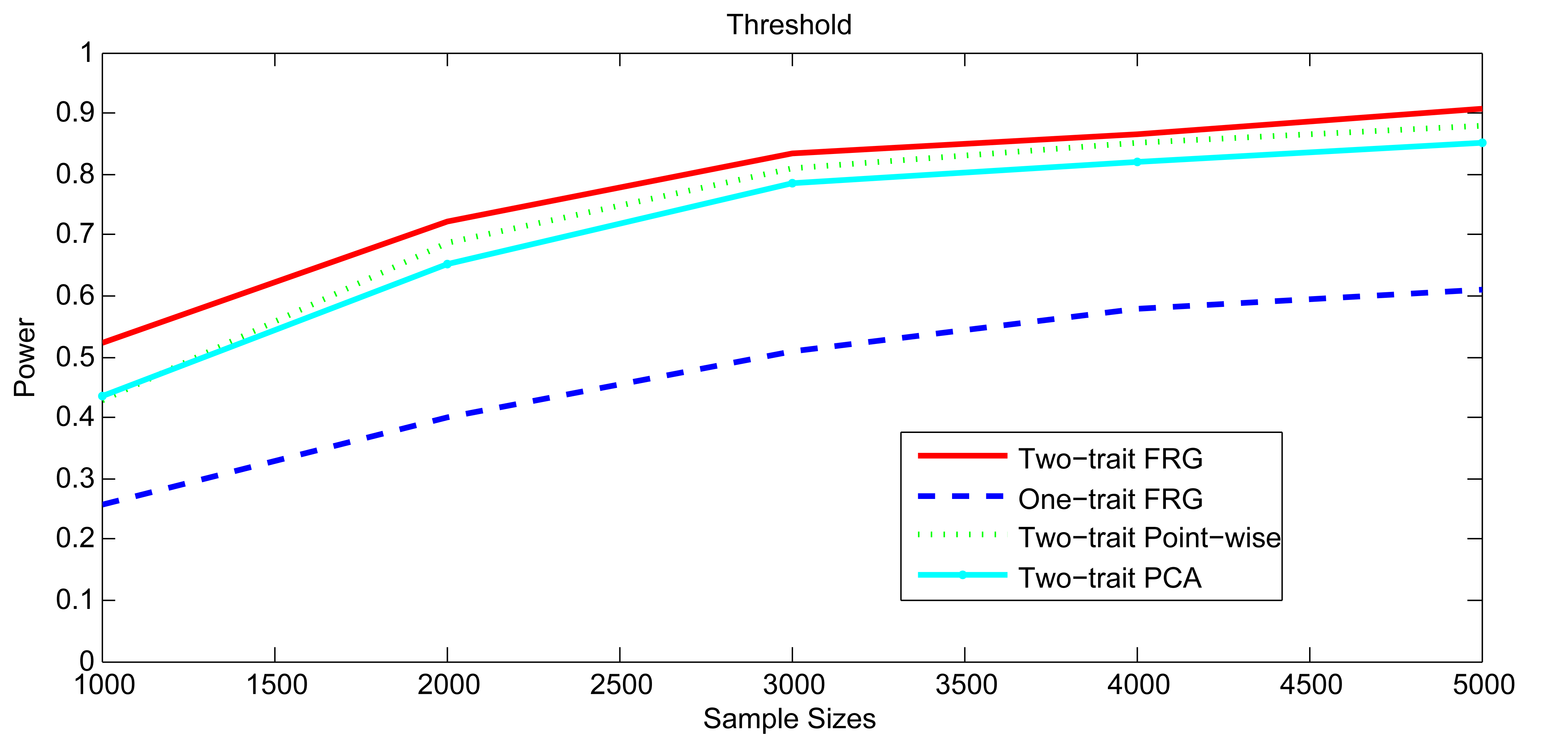

Supplement: S7 Fig — (TIF) [file pgen.1005965.s007.TIF]

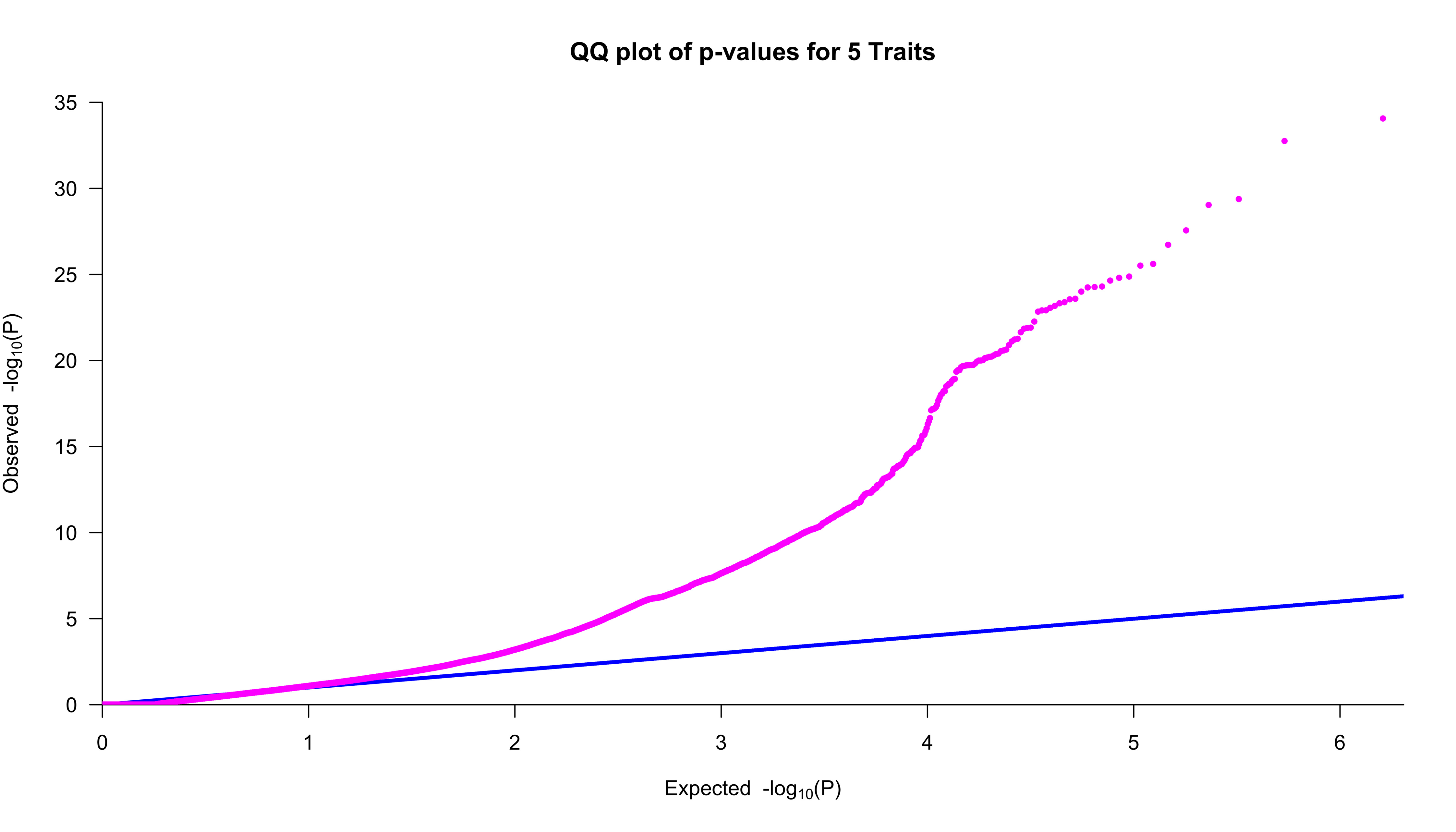

Supplement: S8 Fig — (TIF) [file pgen.1005965.s008.TIF]
