# Supplementary material for: Functional Regression Models for Epistasis Analysis of Multiple Quantitative Traits
Source: PLoS Genet. 2016 Apr 22;12(4):e1005965. doi: 10.1371/journal.pgen.1005965 (PMC4841563; doi:10.1371/journal.pgen.1005965)
Supplement: S1 Table — (DOCX) [file pgen.1005965.s009.docx]

Table S1. Average type 1 error rates of the statistic for testing interaction between two genes with no marginal effect consisting only rare variants with 2 traits over randomly selected 50,000 pairs of genes from the whole exome.

| Sample Size | 0.05 | 0.01 | 0.001 |
| --- | --- | --- | --- |
| 1000 | 0.0578 | 0.0124 | 0.0012 |
| 2000 | 0.0532 | 0.0105 | 0.0012 |
| 3000 | 0.0509 | 0.0112 | 0.0008 |
| 4000 | 0.0494 | 0.0104 | 0.0010 |
| 5000 | 0.0497 | 0.0105 | 0.0011 |
