# Supplementary material for: Functional Regression Models for Epistasis Analysis of Multiple Quantitative Traits
Source: PLoS Genet. 2016 Apr 22;12(4):e1005965. doi: 10.1371/journal.pgen.1005965 (PMC4841563; doi:10.1371/journal.pgen.1005965)
Supplement: S2 Table — (DOCX) [file pgen.1005965.s010.docx]

Table S2. Average type 1 error rates of the statistic for testing interaction between two genes with no marginal effect consisting only rare variants with 10 traits over randomly selected 50,000 pairs of genes from the whole exome.

| Sample size | 0.05 | 0.01 | 0.001 |
| --- | --- | --- | --- |
| 1000 | 0.0735 | 0.0178 | 0.0025 |
| 2000 | 0.0562 | 0.0122 | 0.0017 |
| 3000 | 0.0489 | 0.0099 | 0.0011 |
| 4000 | 0.0487 | 0.0095 | 0.0011 |
| 5000 | 0.0469 | 0.009 | 0.0009 |
