# Supplementary material for: Functional Regression Models for Epistasis Analysis of Multiple Quantitative Traits
Source: PLoS Genet. 2016 Apr 22;12(4):e1005965. doi: 10.1371/journal.pgen.1005965 (PMC4841563; doi:10.1371/journal.pgen.1005965)
Supplement: S3 Table — (DOCX) [file pgen.1005965.s011.docx]

Table S3. Average type 1 error rates of the statistic for testing interaction between two genes with marginal effect consisting only rare variants with 2 traits over randomly selected 50,000 pairs of genes from the whole exome.

| Sample Size | 0.05 | 0.01 | 0.001 |
| --- | --- | --- | --- |
| 1000 | 0.0566 | 0.0127 | 0.0017 |
| 2000 | 0.0523 | 0.0101 | 0.0011 |
| 3000 | 0.0502 | 0.0095 | 0.001 |
| 4000 | 0.0505 | 0.0108 | 0.0011 |
| 5000 | 0.0504 | 0.0103 | 0.0011 |
