# Supplementary material for: Functional Regression Models for Epistasis Analysis of Multiple Quantitative Traits
Source: PLoS Genet. 2016 Apr 22;12(4):e1005965. doi: 10.1371/journal.pgen.1005965 (PMC4841563; doi:10.1371/journal.pgen.1005965)
Supplement: S7 Table — (DOCX) [file pgen.1005965.s015.docx]

Table S7. Average type 1 error rates of the statistic for testing interaction between two genes with marginal effects at two genes consisting only common variants with 2 traits over randomly selected 50,000 pairs of genes from the whole exome.

| Sample size | 0.05 | 0.01 | 0.001 |
| --- | --- | --- | --- |
| 1000 | 0.0508 | 0.011 | 0.0012 |
| 2000 | 0.0491 | 0.0094 | 0.0011 |
| 3000 | 0.0480 | 0.0093 | 0.0008 |
| 4000 | 0.0480 | 0.0093 | 0.0009 |
| 5000 | 0.0489 | 0.0095 | 0.0008 |
