# Supplementary material for: Functional Regression Models for Epistasis Analysis of Multiple Quantitative Traits
Source: PLoS Genet. 2016 Apr 22;12(4):e1005965. doi: 10.1371/journal.pgen.1005965 (PMC4841563; doi:10.1371/journal.pgen.1005965)
Supplement: S9 Table — (DOCX) [file pgen.1005965.s017.docx]

Table S9. The interaction models: 0 and r stand for a quantitative trait mean given the genotypes.

| Models | First locus | Second locus | | |
| --- | --- | --- | --- | --- |
|  |  | $Q_{h_{2}}Q_{h_{2}}$ | $Q_{h_{2}}q_{h_{2}}$ | $q_{h_{2}}q_{h_{2}}$ |
| DominantOR Dominant | $Q_{h_{1}}Q_{h_{1}}$ | r | r | r |
|  | $Q_{h_{1}}q_{h_{1}}$ | r | r | r |
|  | $q_{h_{1}}q_{h_{1}}$ | r | r | 0 |
| DominantAND Dominant | $Q_{h_{1}}Q_{h_{1}}$ | r | r | 0 |
|  | $Q_{h_{1}}q_{h_{1}}$ | r | r | 0 |
|  | $q_{h_{1}}q_{h_{1}}$ | 0 | 0 | 0 |
| Recessive OR Recessive | $Q_{h_{1}}Q_{h_{1}}$ | r | r | r |
|  | $Q_{h_{1}}q_{h_{1}}$ | r | 0 | 0 |
|  | $q_{h_{1}}q_{h_{1}}$ | r | 0 | 0 |
| Threshold | $Q_{h_{1}}Q_{h_{1}}$ | r | r | 0 |
|  | $Q_{h_{1}}q_{h_{1}}$ | r | 0 | 0 |
|  | $q_{h_{1}}q_{h_{1}}$ | 0 | 0 | 0 |
