# Supplementary material for: Functional Regression Models for Epistasis Analysis of Multiple Quantitative Traits
Source: PLoS Genet. 2016 Apr 22;12(4):e1005965. doi: 10.1371/journal.pgen.1005965 (PMC4841563; doi:10.1371/journal.pgen.1005965)
Supplement: S1 Text — (DOCX) [file pgen.1005965.s021.docx]

**Appendix**

**Estimation of Interaction Effects**

We assume that both phenotypes and genotype profiles are centered. The genotype profiles $x_{i}(t)\text{ and }x_{i}(s)$are expanded in terms of the orthonormal basis function as:

$x_{i}(t)=\sum_{j=1}^{\infty} \xi_{ij}\varphi_{j}(t)$and

$x_{i}(s)=\sum_{l=1}^{\infty} \eta_{il}\psi_{l}(s)$’ (A1)

where$\varphi_{j}(t)$and $\psi_{l}(s)$are sequences of the orthonormal basis functions. The expansion coefficients $\xi_{ij}$and $\eta_{il}$ are estimated by

$\xi_{ij}=\int_{T} x_{i}(t)\varphi_{j}(t)dt\text{ }$and

$\eta_{il}=\int_{S} x_{i}(s)\psi_{l}(s)ds$. (A2)

In practice, numerical methods for the integral will be used to calculate the expansion coefficients. Substituting equation (A1) into equation (2) in the text, we obtain

$\begin{matrix} y_{ik}=\alpha_{0k}+\sum_{d=1}^{D} \nu_{id}\tau_{kd}+\int_{T} \alpha_{k}(t)\sum_{j=1}^{\infty} \xi_{ij}\varphi_{j}(t)dt+\int_{S} \beta_{k}(s)\sum_{l=1}^{\infty} \eta_{il}\psi_{l}(s)ds \\ \begin{matrix} & =+ \end{matrix}\int_{T} \int_{S} \gamma(t,s)(\sum_{j=1}^{\infty} \xi_{ij}\varphi_{j}(t))(\sum_{l=1}^{\infty} \eta_{il}\psi_{l}(s))dtds+\varepsilon_{i} \\ \begin{matrix} & =\alpha_{0k}+\sum_{d=1}^{D} \nu_{id}\tau_{kd}+\sum_{j=1}^{\infty} \xi_{ij}\int_{T} \alpha_{k}(t)\varphi_{j}(t)dt+\sum_{l=1}^{\infty} \eta_{il}\int_{S} \beta_{k}(s)\psi_{l}(s)ds \end{matrix} \\ \begin{matrix} & =+\sum_{j=1}^{\infty} \sum_{l=1}^{\infty} \xi_{ij}\eta_{il}\int_{T} \int_{S} \gamma_{k}(t,s)\varphi_{j}(t)\psi_{l}(s)dtds+\varepsilon_{ik} \end{matrix} \\ \begin{matrix} & = \end{matrix}\alpha_{0k}+\sum_{d=1}^{D} \nu_{id}\tau_{kd}+\sum_{j=1}^{\infty} \xi_{ij}\alpha_{kj}+\sum_{l=1}^{\infty} \eta_{il}\beta_{kl}+\sum_{j=1}^{\infty} \sum_{l=1}^{\infty} \xi_{ij}\eta_{il}\gamma_{kjl}+\varepsilon_{ik},i=1,...,n,k=1,...,K, \end{matrix}$(A3)

where $\alpha_{kj}=\int_{T} \alpha_{k}(t)\varphi_{j}(t)dt$, $\beta_{kl}=\int_{S} \beta_{k}(s)\psi_{l}(s)ds$, and $\gamma_{kjl}=\int_{T} \int_{S} \gamma_{k}(t,s)\varphi_{j}(t)\psi_{l}(s)dtds$. The parameters $\alpha_{kj},\beta_{kl}$and $\gamma_{kjl}$are referred to as genetic additive and additive $\times$additive effect scores for the $k$-th trait. These scores can also be viewed as the expansion coefficients of the genetic effect functions with respect to orthonormal basis functions:

$\alpha_{k}(t)=\sum_{j} \alpha_{kj}\varphi_{j}(t),\beta_{k}(s)=\sum_{l} \beta_{kl}\psi_{l}(s)\text{ and }\gamma_{k}(s,t)=\sum_{j} \sum_{l} \gamma_{kjl}\varphi_{j}(s)\psi_{l}(t)$. (A4)

Let

$Y=[Y_{1},...,Y_{K}]=\left[\begin{matrix} Y_{11} & \cdots& Y_{1K} \\ \vdots& \ddots& \vdots\\ Y_{n1} & \cdots& Y_{nK} \end{matrix}\right]$, $e=\left[\begin{matrix} 1 \\ \vdots\\ 1 \end{matrix}\right],\alpha_{0}=\left[\begin{matrix} \alpha_{01} & \cdots& \alpha_{0K} \end{matrix}\right]$, $\nu=\left[\begin{matrix} \nu_{11} & \cdots& \nu_{1D} \\ \vdots& \ddots& \vdots\\ \nu_{n1} & \cdots& \nu_{nD} \end{matrix}\right]$, $\tau_{k}=\left[\begin{matrix} \tau_{k1} \\ \vdots\\ \tau_{kD} \end{matrix}\right],\tau=\left[\begin{matrix} \tau_{1} & \cdots& \tau_{K} \end{matrix}\right]$, $\xi=\left[\begin{matrix} \xi_{11} & \cdots& \xi_{1J} \\ \vdots& \ddots& \vdots\\ \xi_{n1} & \cdots& \xi_{nJ} \end{matrix}\right],\eta=\left[\begin{matrix} \eta_{11} & \cdots& \eta_{1L} \\ \vdots& \ddots& \vdots\\ \eta_{n1} & \cdots& \eta_{nL} \end{matrix}\right]$,$\xi_{i}=\left[\begin{matrix} \xi_{i1} \\ \vdots\\ \xi_{iJ} \end{matrix}\right]$, $\eta_{i}=\left[\begin{matrix} \eta_{i1} \\ \vdots\\ \eta_{iL} \end{matrix}\right]$,$\Gamma=\left[\begin{matrix} \xi_{1}^{T}\otimes\eta_{1}^{T} \\ \vdots\\ \xi_{n}^{T}\otimes\eta_{n}^{T} \end{matrix}\right]=\left[\begin{matrix} \begin{matrix} \xi_{11}\eta_{11} & \cdots& \xi_{11}\eta_{1L} \end{matrix} & \cdots& \begin{matrix} \xi_{1J}\eta_{11} & \cdots& \xi_{1J}\eta_{1L} \end{matrix} \\ \begin{matrix} \cdots& \cdots& \cdots\end{matrix} & \cdots& \begin{matrix} \cdots& \cdots& \cdots\end{matrix} \\ \begin{matrix} \xi_{n1}\eta_{n1} & \cdots& \xi_{n1}\eta_{nL} \end{matrix} & \cdots& \begin{matrix} \xi_{nJ}\eta_{n1} & \cdots& \xi_{nJ}\eta_{nL} \end{matrix} \end{matrix}\right]$,$\gamma_{k}=\left[\begin{matrix} \begin{matrix} \gamma_{k11} \\ \vdots\\ \gamma_{k1L} \end{matrix} \\ \begin{matrix} \gamma_{k21} \\ \vdots\\ \gamma_{k2L} \end{matrix} \\ \begin{matrix} \vdots\\ \begin{matrix} \gamma_{kJ1} \\ \vdots\\ \gamma_{kJL} \end{matrix} \end{matrix} \end{matrix}\right]$,

$\gamma=\left[\begin{matrix} \gamma_{1} & \cdots& \gamma_{K} \end{matrix}\right]$,$\alpha_{k}=\left[\begin{matrix} \alpha_{k1} \\ \vdots\\ \alpha_{kJ} \end{matrix}\right],\alpha=[\alpha_{1},...,\alpha_{K}],\beta_{k}=\left[\begin{matrix} \beta_{k1} \\ \vdots\\ \beta_{kL} \end{matrix}\right],\beta=[\beta_{1},...,\beta_{K}]$,$\varepsilon=\left[\begin{matrix} \varepsilon_{11} & \cdots& \varepsilon_{1K} \\ \cdots& \cdots& \cdots\\ \varepsilon_{n1} & \cdots& \varepsilon_{nK} \end{matrix}\right]$.

Then, equation (A3) can be approximated by

$\begin{matrix} Y=e\alpha_{0}+\nu\tau+\xi\alpha+\eta\beta+\Gamma\gamma+\varepsilon\\ \begin{matrix} & =WB+\varepsilon, \end{matrix} \end{matrix}$ (A5)

where $W=\left[\begin{matrix} \begin{matrix} e & \nu\end{matrix} & \xi& \eta& \Gamma\end{matrix}\right]$and $B=\left[\begin{matrix} \begin{matrix} \alpha_{0} \\ \tau\end{matrix} \\ \alpha\\ \beta\\ \gamma\end{matrix}\right]$.

The standard least square estimators of B and the variance covariance matrix $\Sigma$ are, respectively, given by

$\hat{B}=(W^{T}W)^{-1}W^{T}Y$, (A6)

$\hat{\Sigma}=\frac{1}{n}(Y-WB)^{T}(Y-WB)$. (A7)

Denote the last $JL$row of the matrix $(W^{T}W)^{-1}W^{T}$by $A$. Then, the estimator of the parameter $\gamma$is given by

$\hat{\gamma}=AY$. (A8)

The vector of the matrix $\gamma$can be written as

$vec(\hat{\gamma})=(I\otimes A)vec(Y-\bar{Y})$. (A9).

By the assumption of the variance matrix of $Y$, we obtain the variance matrix of $vec(Y)$:

$\mathrm{var}(vec(Y))=\Sigma\otimes I$. (A10)

Thus, it follows from equations (A9) and (A10) that

(A11)
